# Nicotinamide metabolism-related signature and lncRNA regulatory network in kidney renal clear cell carcinoma

**DOI:** 10.7717/peerj.21300

**Published:** 2026-06-09

**Authors:** Pei Dong, Lincong Zhao, Peiyao Shi, Ming Ma, Beixuan Huang, Zhenda Wang, Shaowei Zhang, Huan Liu

**Affiliations:** The Fourth Hospital of Hebei Medical University, State Grid Hebei Information & Telecommunication Company, Shijiazhuang, Hebei, China

**Keywords:** lncRNA, KIRC, NMRGs, Cox, miRNA

## Abstract

Renal carcinoma ranks among the most lethal cancers, primarily attributed to its atypical symptoms and high metastatic potential. The metabolism of nicotinamide (NAM) plays a significant role in the progression of various tumors. However, investigations into the impact of the NAM metabolism-related signature (NMRS) in kidney renal clear cell carcinoma (KIRC) remain insufficient. Clinical and RNA sequencing data (RNA-seq) from 541 KIRC tissues and 72 adjacent normal tissues were extracted from TCGA. NAM metabolism-related genes (NMRGs) were identified through the Molecular Signatures Database. Cox regression analyses (univariate/multivariate) were conducted to develop NMRS. Time-dependent ROC curve analysis showed that this method had good accuracy in predicting 1-year (AUC = 0.691), 3-year (AUC = 0.749), and 5-year (AUC = 0.764) survival. The Receiver Operating Characteristic (ROC) curve from the external RECA-EU dataset (AUC = 0.682) highlights the accurate prognostic assessment of the NMRS. Overall survival (OS) rates across distinct risk groups were compared using KM analysis. The Cox proportional hazards model assessed the influence of clinicopathological factors and risk scores on survival outcomes (HR = 1.437, 95% CI [1.232–1.676]; *P* < 0.001). Evaluation of the immune microenvironment employed the ESTIMATE and CIBERSORT methods, while enrichment analysis examined biological significance. Correlation analysis determined the link between the expression of checkpoint genes and risk scores. StarBase and miRTarBase facilitated the prediction of target miRNAs and lncRNAs bindingto NMRGs. This study presents a novel framework for identifying prognostic biomarkers and therapeutic targets in KIRC.

## Introduction

Renal cell carcinoma represents the most prevalent genitourinary tumor within urology, with KIRC as its predominant histological subtype. KIRC exhibits significant tumor heterogeneity and distinct pathogenesis (Argentiero et al., 2020). Although the majority of KIRCs exhibit slow growth and limited lethality, approximately 1/3 of patients present with local or distant metastasis at initial diagnosis, indicating unfavorable prognostic outcomes (Zhang et al., 2016; Lipworth, Tarone & McLaughlin, 2011). Surgery remains the primary treatment modality for KIRC (Song, 2017). However, due to its inherent heterogeneity, only a minority of patients derive substantial benefit from targeted therapies.

Consequently, the urgent development of novel and reliable biomarkers is essential for predicting prognosis, tumor progression, and drug sensitivity, thereby facilitating personalized precision therapy.

Nicotinamide (NAM), a water-soluble derivative of Vitamin B3 (niacin), undergoes absorption, metabolism, and excretion in the stomach or intestine, liver, and kidneys, respectively (Surjana, Halliday & Damian, 2010). As a precursor to nicotinamide-adenine dinucleotide (NAD+), NAM plays a significant role in various redox and non-redox reactions that regulate cell’s energy metabolism (Hwang & Song, 2017; Song et al., 2019). Elevated levels of NAD+ (or a high ratio of NAD+/NADH) inhibit the production of ROS and enhance mitochondrial function. A decline in NAD+ associated with cellular aging results in increased ROS levels, which in turn promotes the accumulation of hypoxia-inducible factor-1α, facilitating metabolic reprogramming. NAD deficiency contributes to various kidney diseases (Ralto, Rhee & Parikh, 2020), including AKI (Poyan Mehr et al., 2018; Tran et al., 2016; Jia et al., 2021; Morevati et al., 2021; Doke et al., 2023; Raines et al., 2021; Faivre et al., 2021), diabetic kidney disease (Yasuda et al., 2021; Myakala et al., 2023), adenine-induced kidney disease (Kumakura et al., 2021), and adriamycin-induced focal segmental glomerulosclerosis (Hasegawa et al., 2022). Meanwhile, studies have shown that NAD+ has an effect on tumor proliferation and invasion. He et al. (2022) reported that lncRNA (MDHDH) involved in NAD+ regulation can independently predict OS and therapeutic value in patients with glioblastoma multiforme. This backdrop has prompted investigations into the anti-tumor properties of NAM. In oncological contexts, NAM has demonstrated good effects on non-melanoma skin cancer and mitigates radioresistance induced by tumor hypoxia (Chen et al., 2015; Kaanders, Bussink & Van der Kogel, 2002; Tharmalingham & Hoskin, 2019). Preclinical studies in melanoma models indicate that NAM inhibits angiogenesis, correlating with poor prognostic outcomes (Itzhaki et al., 2013). Furthermore, in primary liver cancer, NAM suppresses hepatocellular carcinoma proliferation and promotes apoptosis *in vitro*, primarily through activation of the p53/p21 pathway (Park et al., 2012). The mechanisms of action of nicotinamide metabolism-related genes and related long non-coding RNAs (lncRNAs) in KIRC have not yet been explored.

Thus, this study established a five-gene NMRS according to the TCGA database. The predictive capabilities of the model were then assessed concerning survival outcomes, immunotherapy responses, and the immune landscape. Furthermore, a ceRNA network linked to NAM cell metabolism was identified, offering potential avenues for novel prognostic biomarkers and therapeutic targets for KIRC.

## Material and Methods

### Data sources

On September 1, 2024, clinical and TPM RNA sequencing data (RNA-seq) from 541 KIRC tissues and 72 adjacent normal tissues were extracted from TCGA (https://portal.gdc.cancer.gov/). Patients for whom clinical information could not be accessed were deleted from subsequent analyses. For our evaluation, data from the International Cancer Genome Consortium (ICGC) project were utilized, specifically the Renal Cell Cancer-European Union (RECA-EU) dataset. This dataset served as an external validation cohort and comprises 91 KIRC samples with available RNA-seq and clinical data (https://www.icgc-argo.org/). Two gene sets linked to NAM metabolism were identified from the molecular signature database (MSigDB) for this study. The study adhered to the guidelines outlined in the Declaration of Helsinki.

### Identification and enrichment analysis of differentially expressed genes

Differentially expressed genes (DEGs) across clusters were identified by the “limma” package, applying the criteria of |log2 fold change (FC)| > 1.5 and false discovery rate (FDR) < 0.05. Subsequently, pathway and functional annotation were conducted *via* Gene Ontology (GO) and Kyoto Encyclopedia of Genes and Genomes (KEGG) enrichment analysis utilizing the R package “clusterProfiler”.

### Construction of predictive NMRGS and validation

A univariate Cox regression analysis identified OS-related NMRGs (*P* < 0.05), followed by a multivariate Cox regression analysis to isolate independent OS-related NMRGs (*P* < 0.05). The expression levels and corresponding coefficients of each prognostic gene informed the calculation of the risk score for KIRC patients: Risk score = (−1.1888 × NMNAT1 expression) + (0.7174 × NADK expression) − (0.6517 × PARP6 expression) − (0.2009 × ENPP3 expression) + (0.2105 × NAMPT expression). As per the median cutoff, KIRC patients were stratified into risk groups for further analysis. The “survival” package was used to conduct the Kaplan–Meier (K–M) survival analysis. Survival curves assessed the capacity of NMRGs to differentiate prognoses across these risk groups. Additionally, the signature’s reliability was evaluated using the “timeROC” package, implementing time-dependent Receiver Operating Characteristic (ROC) curve analysis (Blanche, Dartigues & Jacqmin-Gadda, 2013).

### Development and evaluation of a NAM metabolism-related nomogram

A nomogram was constructed to estimate the 1-, 3-, and 5-year survival probabilities of KIRC patients based on independent predictors. The evaluation of the nomogram’s consistency and accuracy was conducted through calibration and time-dependent ROC curve analyses. Additionally, this nomogram’s net benefit was assessed relative to a model incorporating solely clinical variables *via* decision curve analysis (DCA).

### Mutation of NMRGs

Somatic mutations in maf’ format were from the TCGA database. The “maftools” R package facilitated the creation of waterfall plots, enabling visualization of the mutational landscape in KIRC patients.

### Identification of immune landscape, immunotherapy effect, and drug sensitivity analysis

The ESTIMATE assessed tumor purity, immune, stromal, and ESTIMATE scores. The CIBERSORT utilized the LM22 gene signature matrix to assess the relative proportions of 22 immune cell types in each sample, with 1000 permutations conducted. The relative infiltration of 28 immune cell subsets within the tumor microenvironment of KIRC was quantified through ssGSEA. Drug sensitivity related to NMRGs was evaluated using the GDSC database (http://www.cancerRxgene.org). An FDR value of <0.05 was considered statistically significant.

### Molecular docking and molecular dynamics simulations

We retrieved the structures of the target hub proteins from the Protein Data Bank (PDB; https://www.rcsb) and obtained the structures of the identified active compounds from the PubChem database. These structures were then processed using AutoDock 4.2.6 by removing water molecules and adding hydrogen atoms, followed by conversion to the PDBQT format. Subsequently, we analyzed the binding sites of the target proteins to identify relevant docking pockets. The structural files of both the proteins and key active ingredients were imported into AutoDock Vina 1.2.5 for docking simulations. The resulting output was then visualized as a heatmap to assess the potential binding affinities between the active compounds and the target proteins. Specific docking parameters are as follows: Exhaustiveness (25) and Grid space (0.375). The scoring function uses the default Vina scoring function, and the best docking posture is selected based on the conformation with the highest docking score.

Molecular dynamics simulations were performed using Gromacs 2024.3 (Valdes-Tresanco et al., 2021). Both protein-small molecule systems were configured using OCPLS. Proteins were topologically generated using pdb2gmx, using the Amber99SB-ILDN force field, and small molecules were topologically generated using ACPYPE, using the GAFF2 force field. After merging the topological files, boxes were generated and filled with the TIP3P water model, with NaCl used to neutralize the charge. The system was energy minimized using the steepest descent method, followed by 100 ps of system equilibration and finally a 100 ns molecular dynamics simulation.

### Expression analysis

The expressions of NMRGs at mRNA and protein levels in KIRC were analyzed and confirmed using the UALCAN database (http://ualcan.path.uab.edu/index.html) (Chandrashekar et al., 2017). Additionally, protein expressions were validated through IHC sourced from the HPA database (https://www.proteinatlas.org/).

### Cell culture

Human kidney renal clear cell carcinoma (KIRC) cell lines Caki-1 and 786-O, along with the normal human embryonic kidney cell line 293T, were used in this study. All cell lines were obtained from the Shanghai Institutes for Biological Sciences (Shanghai, China). The 293T cells were cultured in high-glucose Dulbecco’s Modified Eagle Medium (DMEM), while 786-O and Caki-1 cells were maintained in RPMI-1640 and McCoy’s 5a medium (Procell, China), respectively. A supplement of 10% fetal bovine serum (FBS) and 100 U/mL penicillin-streptomycin was added to all media. Cell cultures were maintained at 37^∘^C in a humidified atmosphere of 5% CO_2_.

### RNA extraction and cDNA Synthesis

Total RNA was extracted using TRIzol reagent (Takara, Shiga, Japan) following the manufacturer’s protocol. The concentration and purity of RNA were measured using a NanoDrop spectrophotometer (Thermo Fisher Scientific, Waltham, MA, USA). Samples with an absorbance ratio (A260/A280) between 1.8 and 2.0 were considered to have high purity. One microgram of total RNA was used for cDNA synthesis using the PrimeScript™ RT reagent Kit with gDNA Eraser (Takara, Japan), according to the manufacturer’s instructions. The reverse transcription was performed on a thermal cycler (Bio-Rad, Hercules, CA, USA) under the following conditions: 37 ^∘^C for 30 min, and 85 ^∘^C for 5 s to inactivate the reverse transcriptase.

### Quantitative real-time PCR

Quantitative real-time PCR (qPCR) was conducted using SYBR Green SuperMix (Takara, Shiga, Japan) on an Applied Biosystems 7500 Real-Time PCR System (Applied Biosystems, Foster City, CA, USA). The thermal cycling conditions included an initial denaturation at 95 ^∘^C for 5 min, followed by 40 cycles of denaturation at 95 ^∘^C for 10 s and annealing/extension at 60 ^∘^C for 20 s. A melt curve analysis was performed to confirm amplification specificity. Primers were synthesized by Sangon Biotech (Shanghai, China), and their sequences are listed in Table S1.

### Data normalization and analysis

Gene expression levels were normalized to GAPDH as an internal control. Relative quantification was calculated using the 2^−ΔΔCt^ method. Each sample was analyzed in triplicate, and mean Ct values were used for expression analysis. Primer efficiencies ranged between 90% and 110%, ensuring reliable quantification. GraphPad Prism 8 (GraphPad Software, La Jolla, CA, USA) was employed for all statistical analyses.

### Identification of ceRNA network

The line of gene expression levels to clinical stages was analyzed using GEPIA (http://gepia.cancer-pku.cn/). To investigate the potential role of NMRGs in KIRC and elucidate their molecular mechanisms, StarBase (https://starbase.sysu.edu.cn/) and miRTarBase (https://miRTarBase.cuhk.edu.cn/) were employed to predict miRNAs that bind to NMRGs. mRNA and miRNA interactions were visualized using Cytoscape (version 3.10.0). The top 10 highly connected mRNAs and miRNAs were identified through the CytoHubba’s MCC method (Cytoscape). Additionally, univariate Cox regression analysis was conducted to identify miRNAs associated with prognosis. Based on these selected miRNAs, lncRNAs interacting with miRNA targets were predicted using StarBase.

### Statistical analysis

Statistical analyses and graph generation utilized R software version 4.1.3. Survival differences among various risk groups were assessed through Kaplan–Meier curves and the log-rank test. The comparison of categorical variables between the NMRGs-high and -low groups employed the Chi-square test. Correlation coefficients were calculated using the Spearman test. A *P* value of <0.05 indicated statistical significance.

## Results

### Genomic and transcriptomic landscape of NMRGs

**Figure 1:**
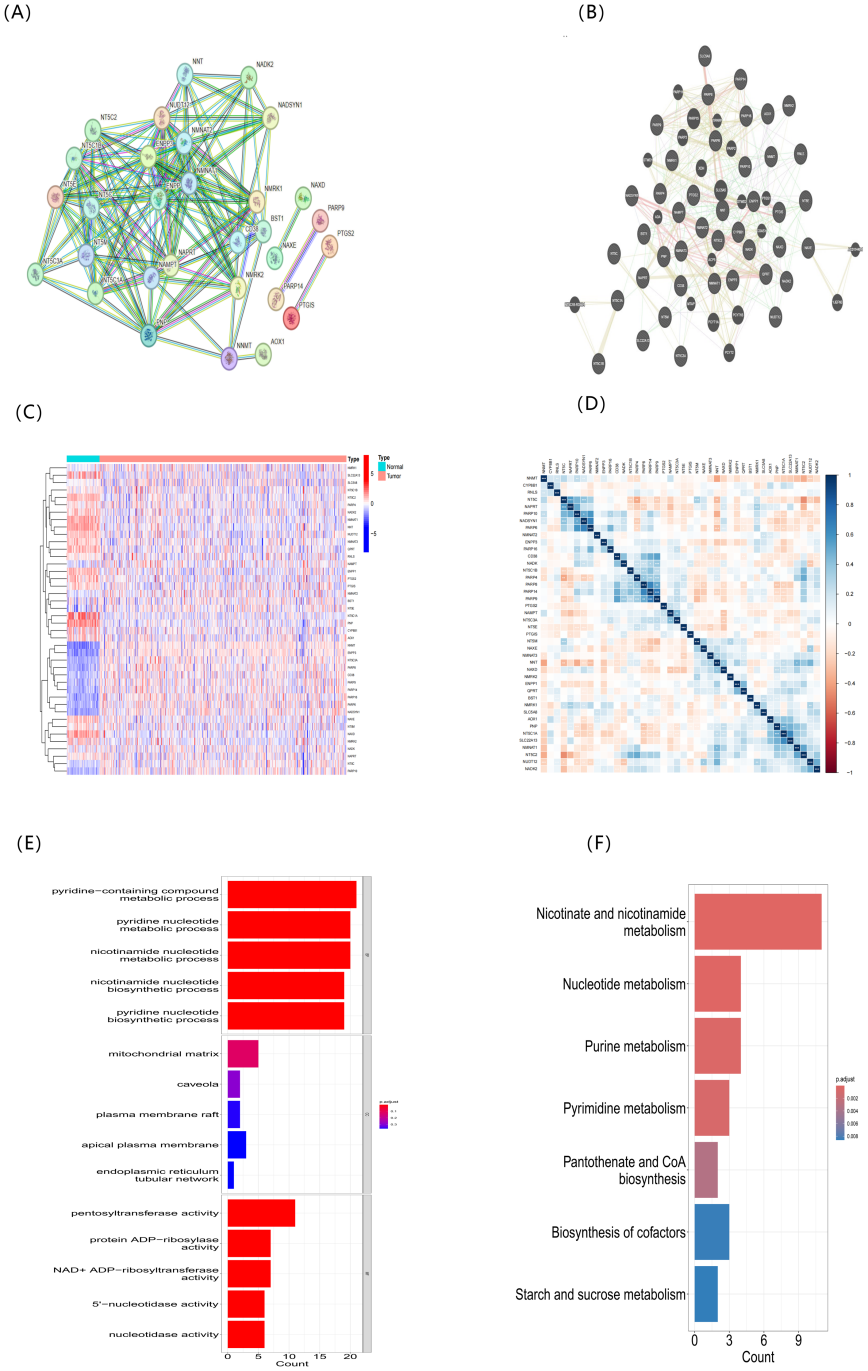
Evaluation of NMRGs in KIRC. (A) PPIs between NMRGs using the STRING database. (B) Analysis of DEGs and their co-expressed genes *via* GeneMANIA. (C)Heatmap displaying the expression of 42 NMRGs in the KIRC and normal tissue samples using the TCGA database; (D) Correlation analysis between 42 NMRGs. (E, F) GO and KEGG pathway analysis of NMRGs.

Using two NAM metabolism-related gene sets, 42 NMRGs were identified (Table S2). A protein-protein interaction (PPI) network was analyzed through the STRING database to assess associations among these NMRGs (Fig. 1A). To clarify the interrelationships among NMRGs, a co-expression network was constructed, followed by an investigation into their associated functions (Fig. 1B). Marked differences in NMRGs expression were observed between normal and KIRC samples (Fig. 1C). The co-expression relationships among the 42 NMRGs were illustrated in Fig. 1D. Additionally, GO and KEGG analyses were conducted based on these 42 NMRGs (Figs. 1E–1F).

### Development of NAM metabolism-related prognostic features

**Figure 2:**
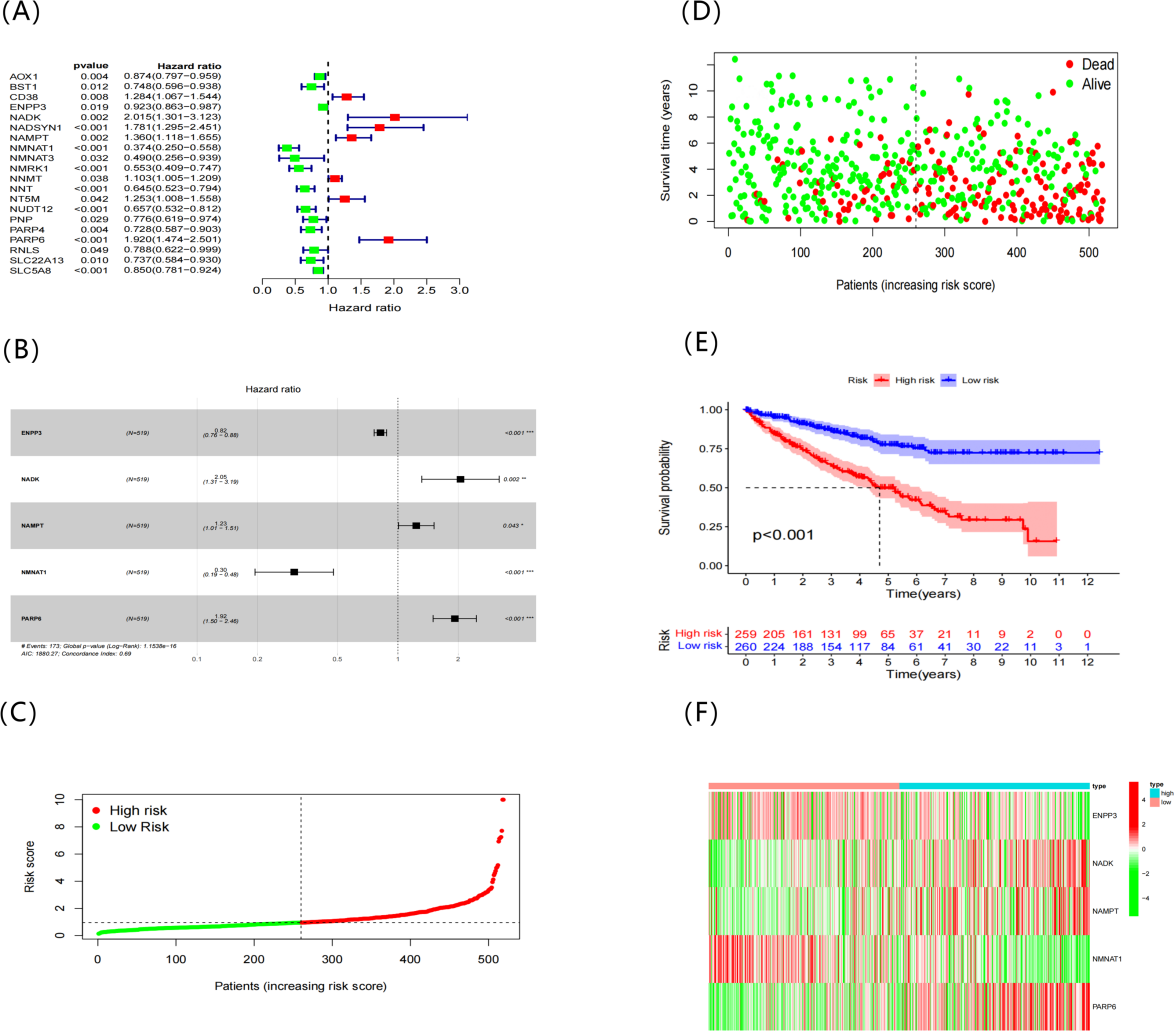
Development and assessment of prognostic signature based NMRGs in the TCGA date set. (A, B) Univariate and multivariate Cox regression analyses were performed for assessing the prognostic values of the NMRGs, with regards to KIRC. (C–E) Risk score distribution (C), survival status of each patient (D), and Kaplan–Meier curves of low- and high-risk subgroups based on risk score (E). (F) Heatmaps of the prognostic 5-gene signature in the TCGA database.

Univariate Cox regression analysis identified NMRGs linked to KIRC using data from the training cohort (Fig. 2A), resulting in the identification of 20 prognostically relevant NMRGs. These genes were subsequently subjected to multivariate Cox regression analysis, revealing five independent predictors of KIRC: three risk factors (*NAMPT*, *NADK*, *PARP6*) and two protective factors (*ENPP3*, *NMNAT1*) (Fig. 2B). The model was formulated based on multivariate Cox regression coefficients and the expressions of these five key genes. The risk score for each KIRC patient was calculated as follows: Risk score = (−1.1888 × NMNAT1 expression) + (0.7174 × NADK expression) − (0.6517 × PARP6 expression) − (0.2009 × ENPP3 expression) + (0.2105 × NAMPT expression). Individual risk scores were established for patients in the TCGA-KIRC cohort, categorizing them into distinct risk groups as per the median value. The distribution of risk scores and corresponding survival statuses was depicted in Figs. 2C–2D. Survival analysis indicated that patients in the low-risk group experienced improved outcomes (Fig. 2E, *P* < 0.001).

The heatmap illustrated the expressions of five key genes across the two NMRGS subgroups (Fig. 2F). To evaluate the prognostic accuracy of these genes and their impact on OS, separate Kaplan–Meier analyses were performed for each gene utilizing the database (Fig. S1).

### Risk score may be an independent predictor of OS

**Figure 3:**
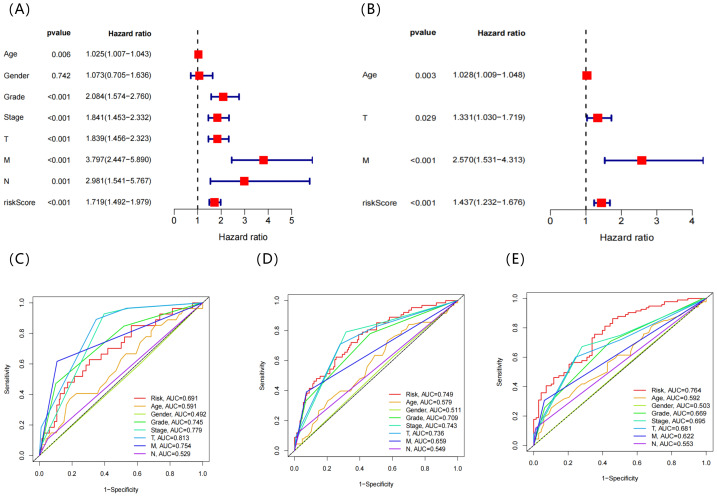
Independent prognostic analysis of risk scores. (A) Prognostic effect analysis of risk score and clinical features in KIRC with univariate Cox regression analysis. (B) Independent prognostic effect analysis of risk score and traditional prognostic clinical features in KIRC with multivariate Cox regression analysis. (C–E) Receiver operating characteristic analysis of KIRC risk scores and other prognostic clinical characteristics to predict the 1-, 3-, and 5-year survival rate of KIRC patients.

Regression analyses (univariate and multivariate) identified risk score as an independent risk factor for survival in TCGA-KIRC (Figs. 3A, 3B). ROC analysis revealed AUC values of 0.691 at one year, 0.749 at three years, and 0.764 at five years, indicating that the risk score possesses robust predictive capability for patient survival. Comparatively, the NMRGs risk score outperformed other clinicopathological factors in predicting survival among KIRC patients (Figs. 3C–3E).

A significant correlation existed between the risk score and the five NMRGs, and clinicopathological factors (Fig. S2). To verify these results, RECA-EU data were obtained from the ICGC database for analysis, which confirmed the differences between risk groups and the validity of the constructed prognosis model (Fig. S3).

### Construction of nomograms based on risk scores and WHO grades

**Figure 4:**
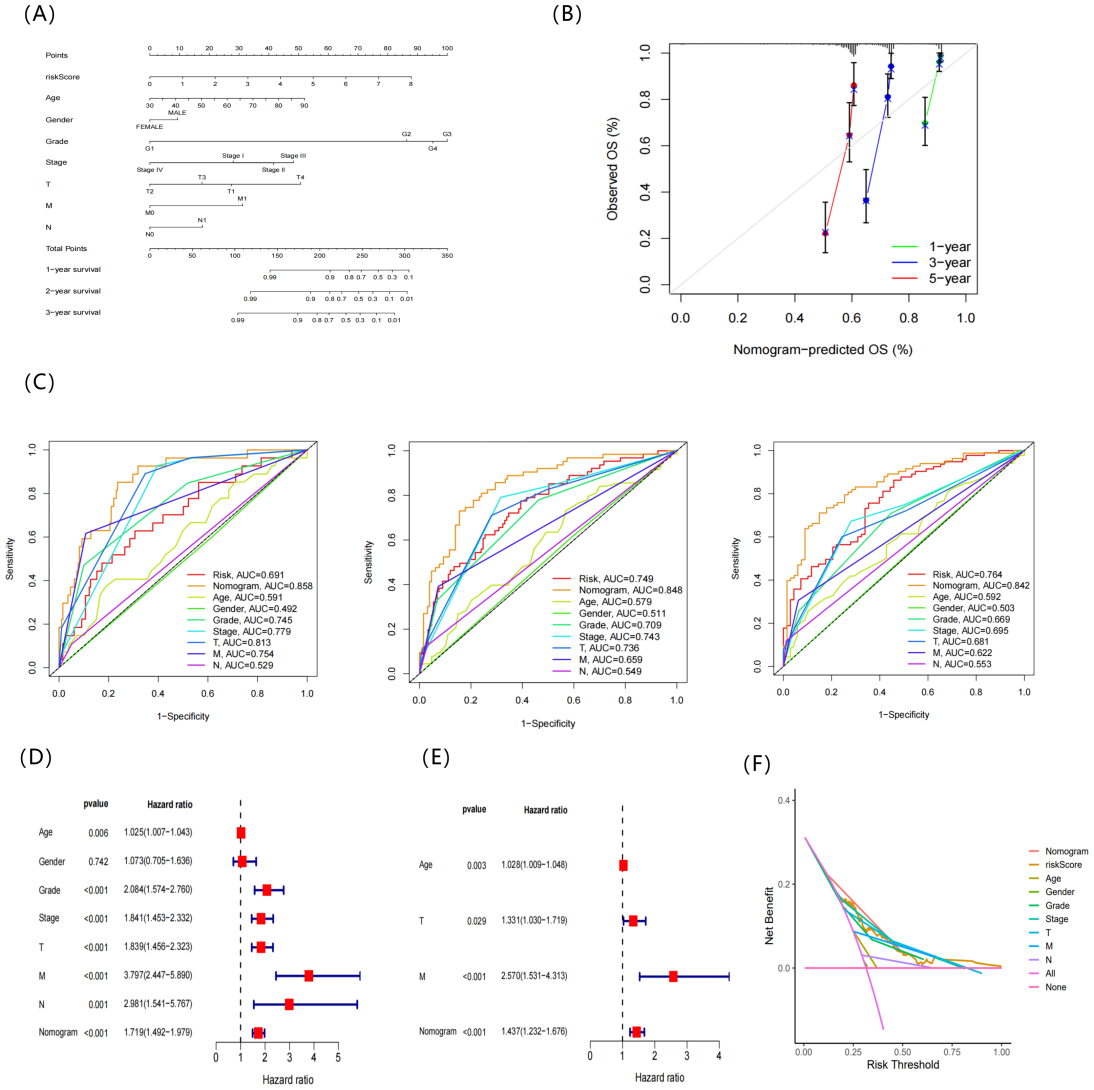
Establishment of a predictive nomogram. (A) Nomogram predicting the 1-, 2- and 3-year survival rates for KIRC patients. (B) Calibration curves for the nomogram prediction validation cohort of KIRC patient survival (*x*-axis: the predicted survival probability; *y*-axis: the actually observed survival probability). (C) Receiver operating characteristic analysis of the KIRC nomogram and other prognostic clinical characteristics to predict the 1-, 3-, and 5-year survival rates of KIRC patients. (D) Assessing the relationship between risk scores and clinical factors with OS using a forest plot of the univariate Cox test results. (E) The independent risk factors for OS were determined using a forest plot of the multivariate Cox analysis results. OS, overall survival; AUC, area under the curve; CI, confidence interval; KIRC, kidney renal clear cell carcinoma. (F) DCA curves of the clinicopathological indicators and this nomogram.

To enhance the clinical utility of this novel risk model, a nomogram incorporating risk scores and clinicopathological features was developed to predict OS at 1, 3, and 5 years (Fig. 4A). Calibration graphs based on the NMRGS nomogram demonstrated strong alignment between actual and predicted probabilities for OS at these time points (Fig. 4B). The AUC values for 1, 3, and 5 years were 0.858, 0.848, and 0.842, respectively, confirming the nomogram’s predictive accuracy (Fig. 4C). Both regression analysis results (multivariate and univariate) identified the nomogram as an independent risk factor for survival (Figs. 4D, 4E). The nomogram’s clinical applicability was further assessed through DCA. Compared to models based solely on clinical characteristics, The comprehensive nomogram was associated with superior net benefits, which may facilitate improved clinical management outcomes (Fig. 4F).

### DEGs, function enrichment analysis, and somatic mutations in two risk groups

**Figure 5:**
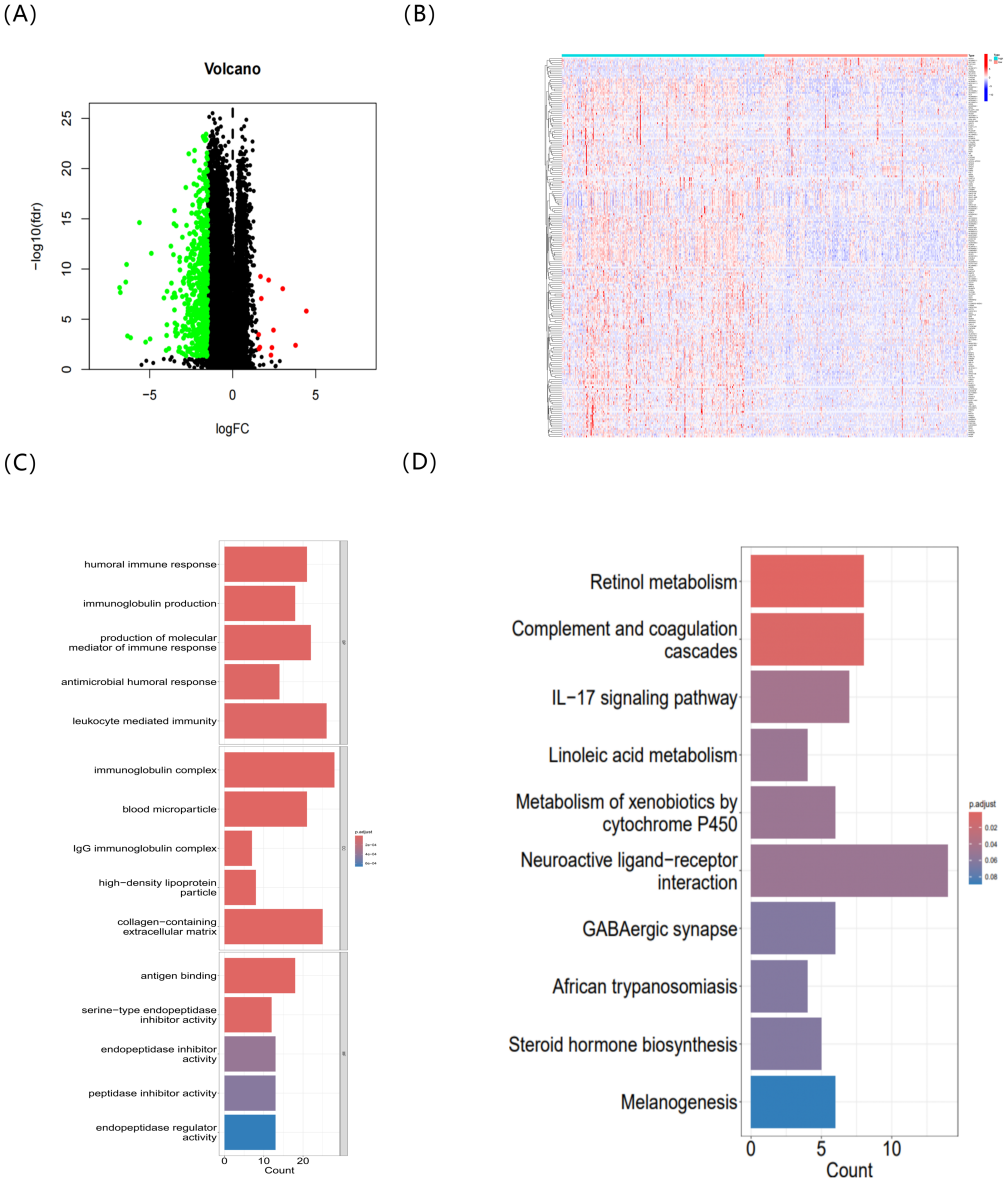
Analysis of the differentially expressed genes (DEGs) and functional enrichment analysis in different NMRGs risk scores. (A) Volcano plot of the distribution of DEGs between the NMRGs-high and NMRGs-low risk groups in the TCGA cohort. (B) Heatmap of DEGs expression between the NMRGs-high and NMRGs-low risk groups. (C, D) The GO and KEGG enrichment analyses of signaling pathways; the color of the dot represents the *p* value.

**Figure 6:**
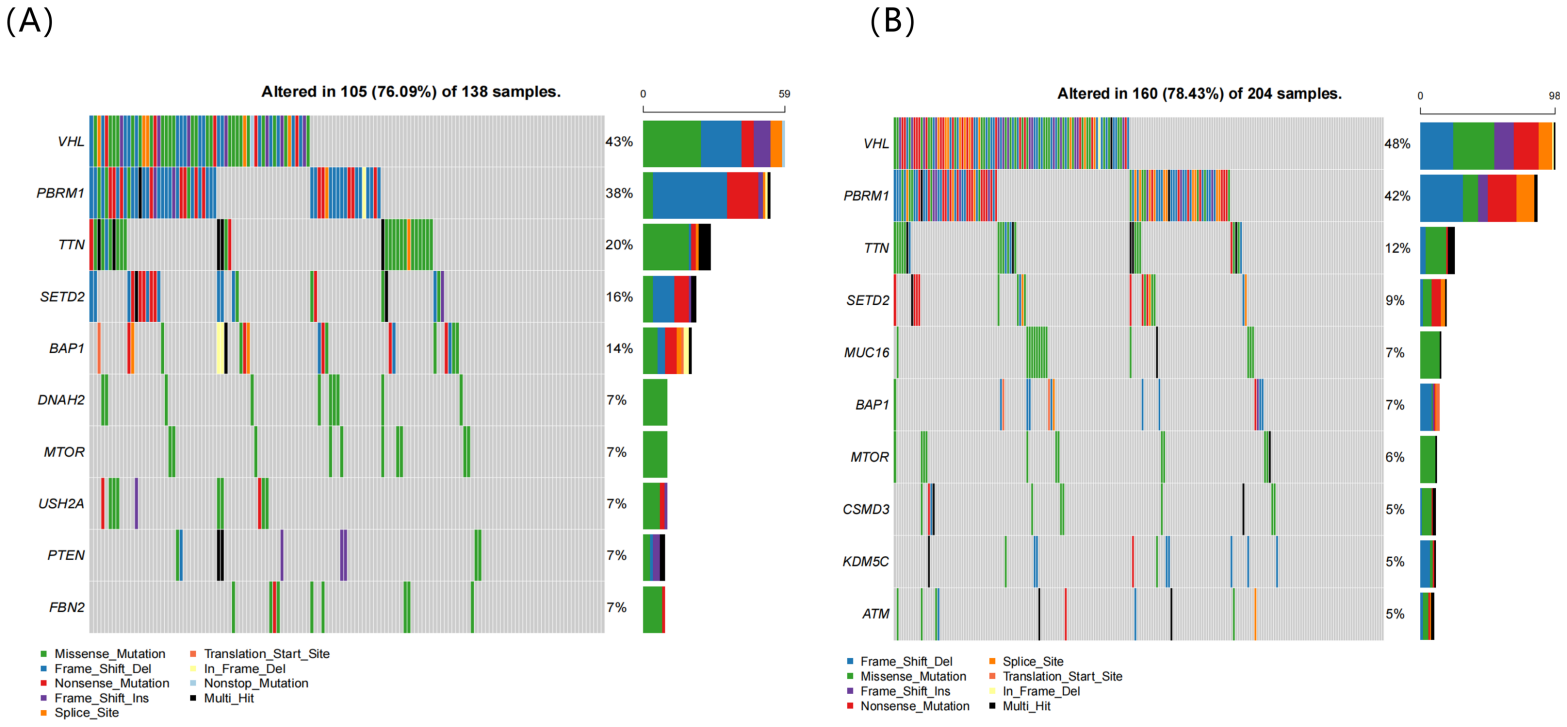
Comparison of somatic mutations between different NMRGs subtypes. (A, B) Oncoprint visualization of the top ten most frequently mutated genes in NMRGs-high subtype (A), and NMRGs-low subtype (B).

The differential analysis identified 592 DEGs between the two groups (Figs. 5A–5B). Analyses of GO and KEGG were conducted on these DEGs (Figs. 5C–5D). Additionally, mutated genes in both risk groups were examined. Figures 6A–6B presented the top 10 genes exhibiting the highest mutation frequencies in each group. Among the high-risk group, 105 out of 138 samples (76.09%) contained mutations, whereas in the low-risk group, 160 out of 204 samples (78.43%) displayed mutations. The five most frequently mutated genes in the high-risk group were VHL, PBRM1, TTN, SETD2, and BAP1, while the low-risk group shared VHL, PBRM1, TTN, SETD2, with MUC16 as the fifth most mutated gene.

### Identification of immune landscape

**Figure 7:**
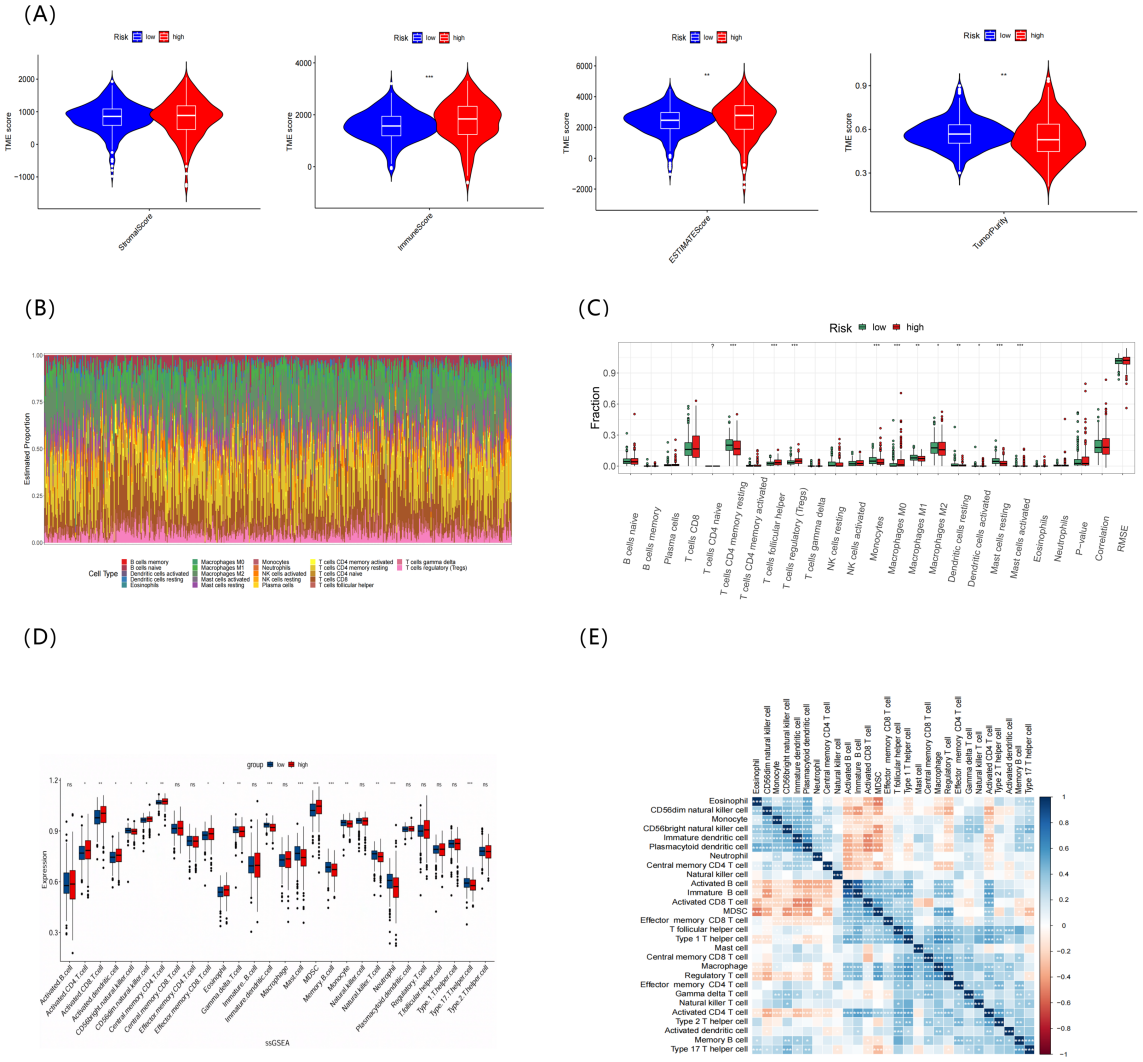
The immune landscape between the NMRGs-high- and NMRGs-low groups. (A) Violin plots of the stromal score, immune score, ESTIMATE score, and tumor purity between the NMRGs-high and NMRGs-low risk groups. (B) Relative percent of immune infiltration in the NMRGs-high and NMRGs-low risk groups. (C) The fraction of 22 immune cells in low- and high-risk group calculated by CIBERSORT algorithm. (D) The fraction of 28 immune cells low- and high-risk group calculated by ssGSEA algorithm. (E) Heatmap of the correlations among different immune cells. Blue and red illustrate positive and negative associations, respectively. ns, *P* > 0.05; *, *P* < 0.05; **, *P* < 0.01; ***, *P* < 0.001.

The ESTIMATE and CIBERSORT algorithms facilitated the analysis of the tumor microenvironment (TME). ESTIMATE analysis revealed that the low-risk group exhibited lower ImmuneScores and ESTIMATEScores, alongside higher TumorPurity (*P* < 0.05) (Fig. 7A). The CIBERSORT algorithm assessed the relative immune cell infiltration scores across 22 immune cell types and summarized the findings for KIRC patients within the TCGA dataset (Fig. 7B). Notably, the low-risk group displayed increased proportions of resting memory CD4 T cells, M1 and M2 macrophages, and resting mast cells. In contrast, higher proportions of follicular helper T cells and regulatory T cells were observed in the high-risk group (Fig. 7C). These results indicate a potential correlation between risk scores and immune infiltration levels, possibly impacting KIRC patients’ prognosis.

Overall, the results suggest that the NMRG risk model correlates with immune infiltration status and reflects the immune profile of KIRC patients.

Additionally, ssGSEA calculated the scores of 28 immune cell types and analyzed their differential expression levels across two risk subgroups (Fig. 7D). Furthermore, the relationships among these 28 immune cells were investigated (Fig. 7E).

**Figure 8:**
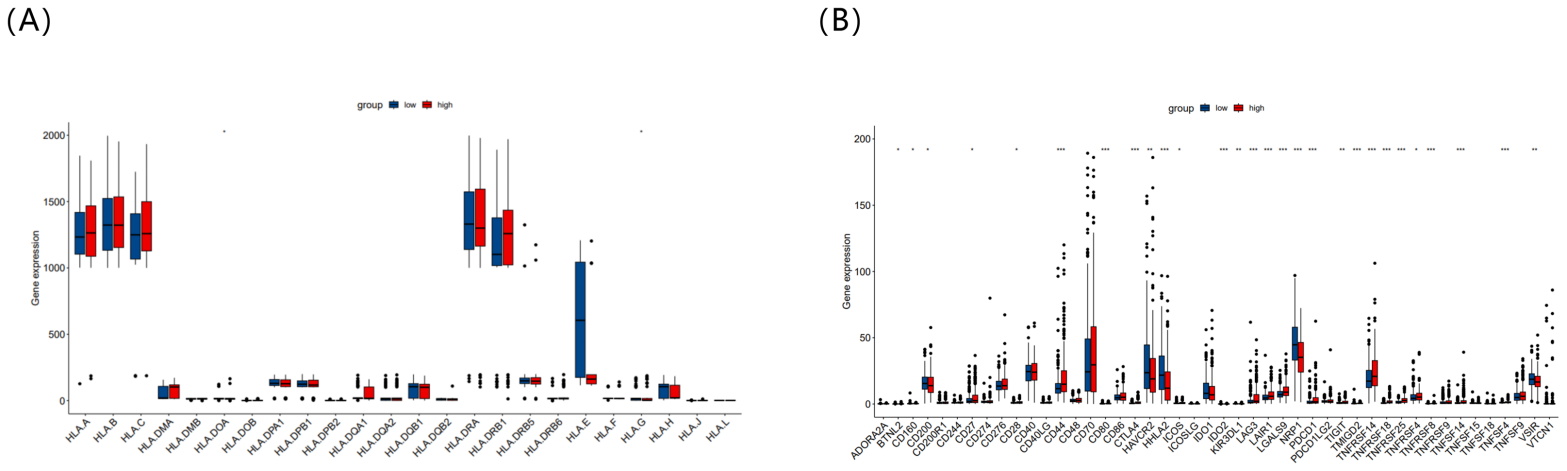
Differential expression of HLA genes and immune checkpoints. Box plots depict the differential expression of HLA genes (A) and immune checkpoints (B) between the NMRGs high-risk group and the NMRGs low-risk group. *, *P* < 0.05; **, *P* < 0.01; ***, *P* < 0.001.

Due to the importance of checkpoint inhibitor immunotherapy, we analyzed the expression of 24 human leukocyte antigen (HLA) genes and 47 immune checkpoints in the two groups. The results indicated that the high-risk group exhibited significantly higher expression levels of molecules such as CD27, CD28, CD44, CD70, LAIR1, LGALS9, TNFRSF14, TNFRSF4, and PDCD1 (Figs. 8A–8B). The upregulation of immune checkpoints and immunosuppressive cytokines appears to induce a suppressed immune microenvironment (IME) in high-risk patients.

### Drug sensitivity analysis

**Figure 9:**
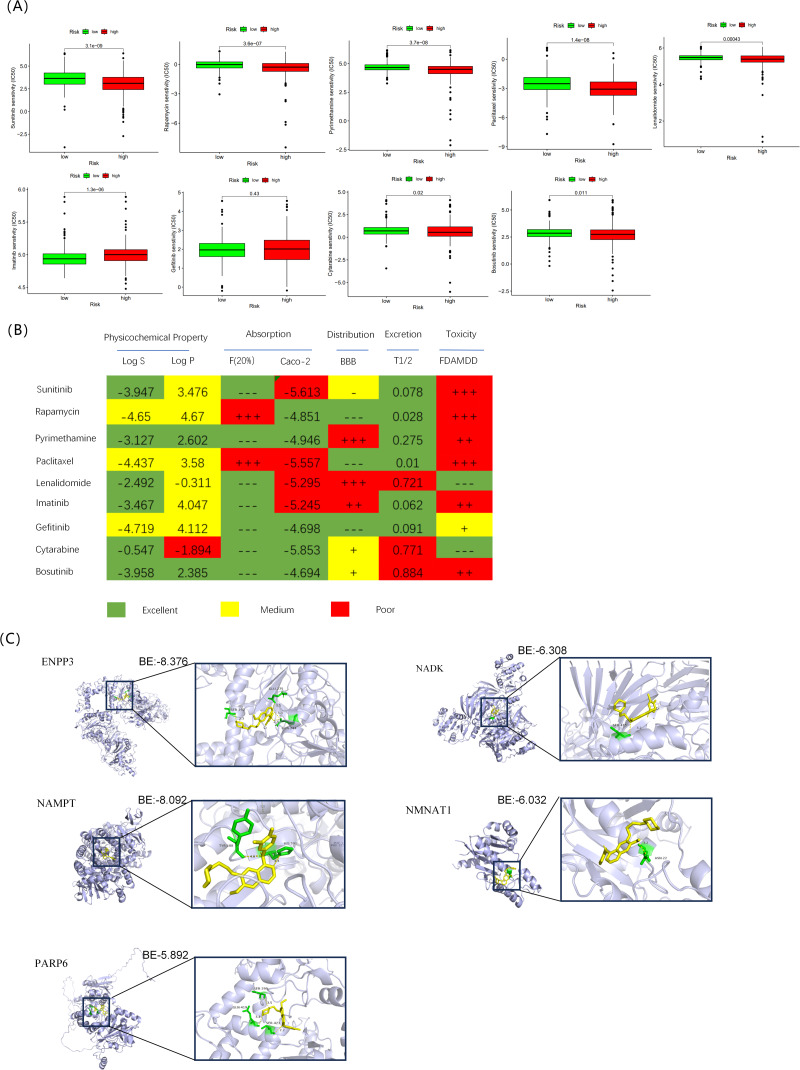
Drug sensitivity analysis in low- and high-risk group. (A) The IC50 value exhibits a significant difference in low- and high-risk group among Sunitinib, Rapamycin, Pyrimethamine Paclitaxel, Lenalidomide, Imatinib, Gefitinib, Cytarabine, and Bosutinib. (B) ADMET attributes of potential therapeutic drugs. (C) Three-dimensional docking patterns, three-dimensional structures and binding patterns showing the interaction bonds formed between the predicted pockets of the Gefitinib compound and the core targets.

The IC50 value served as a sensitivity indicator to evaluate the chemotherapy effects of ten commonly used small-molecule drugs in KIRC patients. Figure 9 illustrated that the IC50 values for sunitinib, rapamycin, paclitaxel, pyrimethamine, lenalidomide cytarabine, and bosutinib were significantly higher in the low-risk group, whereas the IC50 for imatinib was lower (Fig. 9A). Figure S4 illustrated the correlation between the risk score and drug sensitivity. These results indicate that KIRC patients in distinct risk subgroups exhibit varying responses to different anti-tumor agents, highlighting the potential for personalized treatment to significantly benefit patients.

We analyzed the ADMET of these candidate drugs using the data source ADMETlab 2.0 (https://admetmesh.scbdd.com/). The observations suggested that the Gefitinib compound is the most promising drug (Fig. 9B). These candidate drugs have the potential to be therapeutic drugs in KIRC. Molecular docking analysis was performed to examine Gefitinib’s binding affinities with the five NMRGs and assess their potential therapeutic significance. Binding energies less than −5 kcal/mol indicated notable binding affinity, while values below −7.0 kcal/mol suggested a strong binding interaction. Gefitinib forms a hydrogen bond with *ENPP3* and *NAMPT* at specific sites, resulting in a binding energy of −8.376 kcal/mol and −8.092 kcal/mol, reflecting a strong interaction. Higher binding energy values were observed for Gefitinib with *NADK* (−6.308), *NMNAT1* (−6.032), and *PARP6* (−5.892), signifying relatively weak affinities (Fig. 9C).

**Figure 10:**
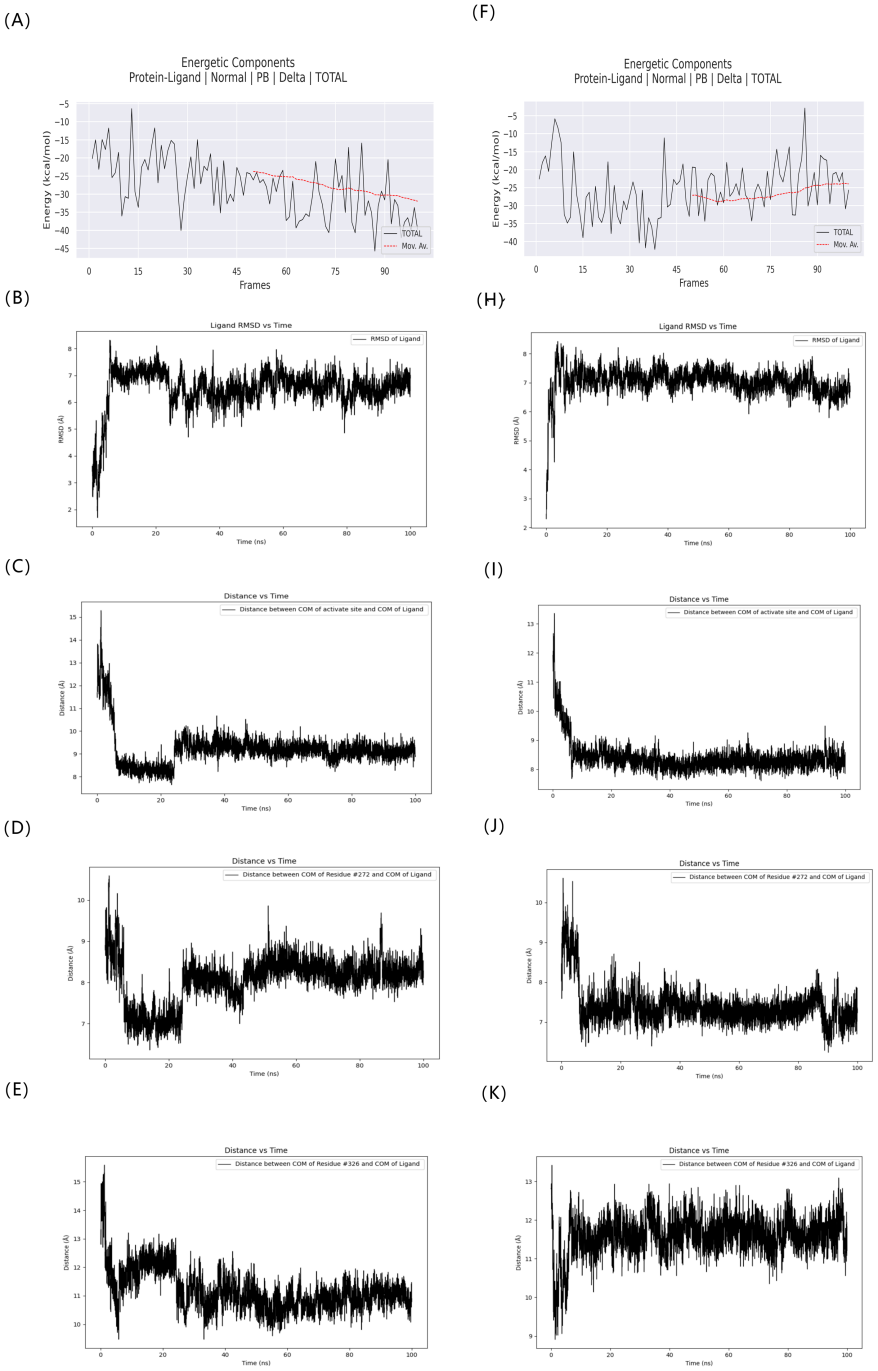
Binding stability. (A, F) Changes of absolute free energy over time (1ns/frame). (B, H) The RMSD of the small molecule changes over time. (C, I) The distance between the center of mass of the active residue and the center of mass of the small molecule changes over timeRMSD. (D–E, J–K) Evolution of ligand features and distances between residues on either side of the active site.

The highest-scoring Gefitinib-*ENPP3* complex was further assessed by molecular dynamics simulations to investigate its binding stability in greater depth. As shown in the Fig. 10A, the absolute free energy changes with time and remains at −30 kcal/mol at 90 ns.

The binding stability was further evaluated by root mean square deviation (RMSD) analysis. Throughout the process, the RMSD of the small molecule remained basically stable after stabilization, without obvious conformational changes or deviations from the active site (Fig. 10B).

By analyzing the distance between the center of mass of the active site and the center of mass of the small molecule, it was found that there was no obvious distance or proximity during the entire process after stabilization, which proved the stability of the binding of small molecules to proteins (Fig. 10C).

We selected two residues on either side of the active site and analyzed the evolution of ligand characteristics and the distance between the two residues (Figs. 10D–10E). The binding free energies and energy components of this complex are shown in Table S3. The simulation was run again with different initial velocities and the results were largely consistent (Figs. 10F–10K).

### Validation of NMRGs

**Figure 11:**
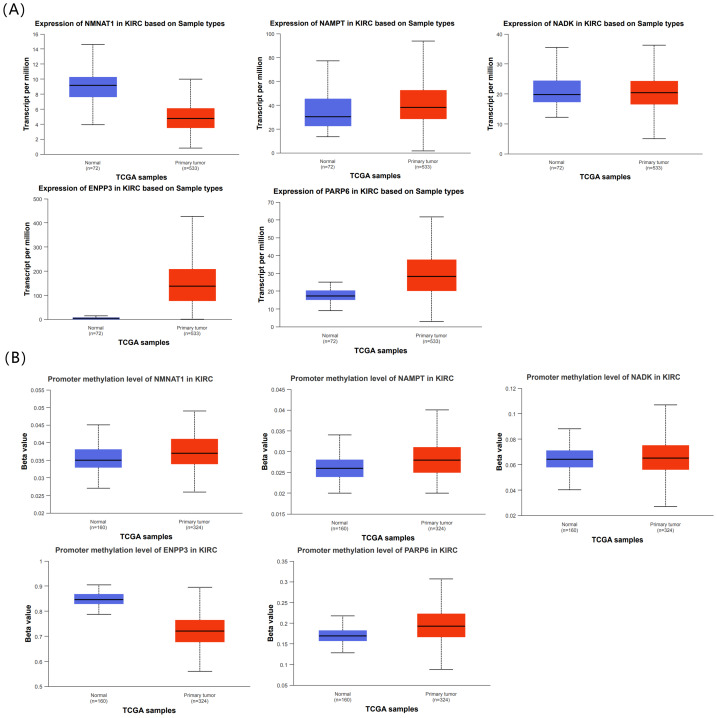
Validation of the mRNA expression levels (A) and promoter methylation levels (B) of NMRGs *via* UALCAN database.

Validation of mRNA and protein expression levels of NMRGs utilized the UALCAN and HPA databases. *NADK* and NMNAT1 exhibited reduced expression in primary tumor tissues relative to normal tissues, whereas other genes such as *ENPP3*, *NAMPT*, and *PARP6*, displayed elevated expression (Fig. 11A). Notably, the methylation levels of the prognostic gene (*ENPP3*, *NADK*, and *NMNAT1*) promoters inversely correlated with gene expression levels (Fig. 11B).

**Figure 12:**
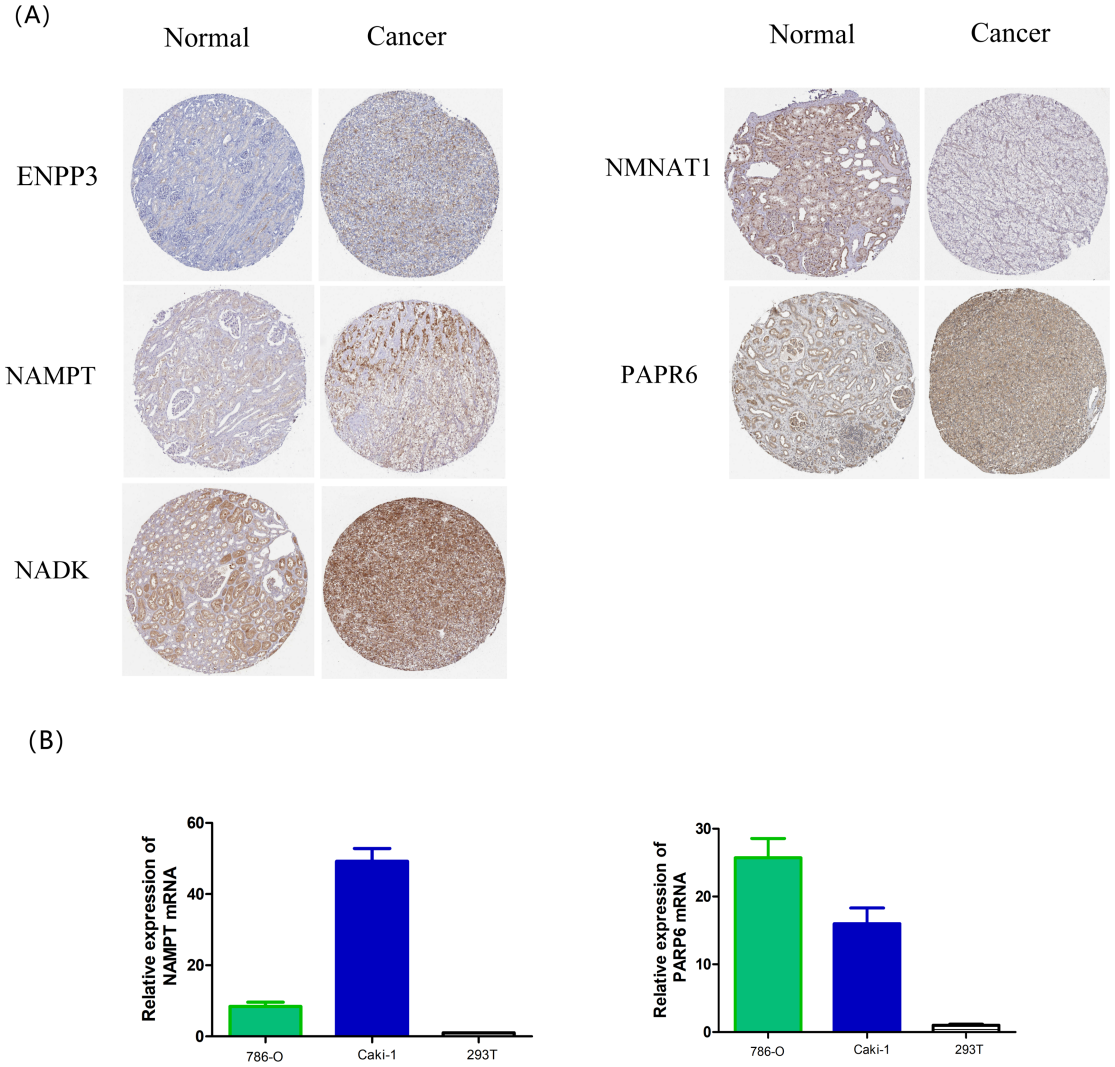
Validation of the protein expression levels of NMRGs genes *via* IHC in HPA database (A). Validation of gene expression levels was performed by comparing tumor cells to normal cells using quantitative RT-PCR (B).

Furthermore, protein expression levels for several prognostic genes were confirmed (Fig. 12A), aligning with the observed gene expression patterns. Finally, we validated the expression and function of NMRGs in cell lines. Consistently, elevated *NAMPT* and *PARP6* levels were observed in KIRC cell lines compared to the normal human 293T cells (Fig. 12B).

### Construction of the ceRNA network

**Figure 13:**
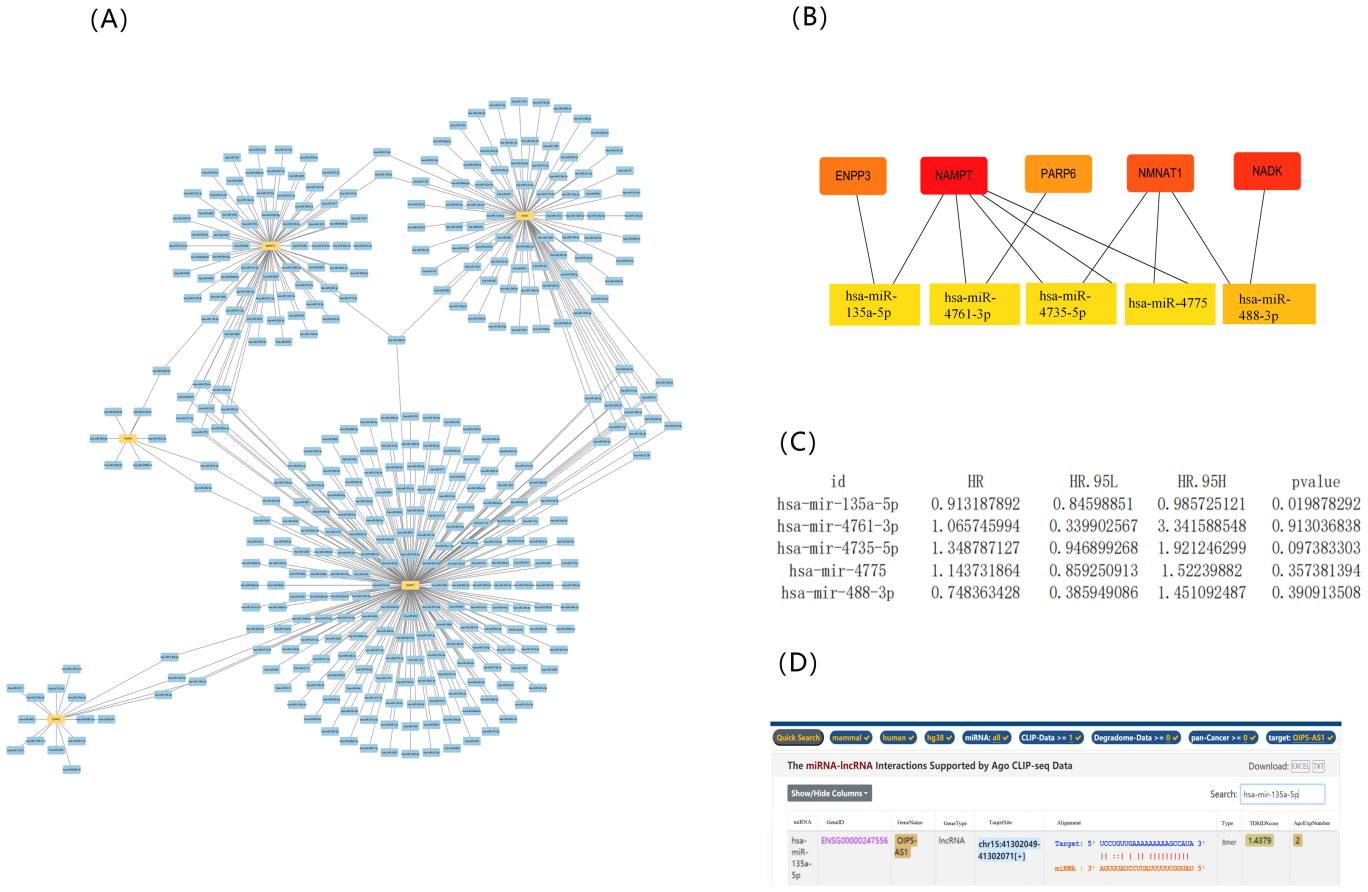
Construction of the ceRNA network. (A) Results of miRNA target predicted by mirTarBase and Starbase. (B) The highly connected target mRNAs calculated by Cytohubba. (C) Univariate cox regression analysis of the highly connected target mRNAs. (D) The expression lncRNA OIP5-AS1 in KIRC. (E) The correlation between survival possibility and the expression of lncRNA OIP5-AS1 showed by Kaplan–Meier analysis.

Analysis revealed that *ENPP3*, *NAMPT*, *PARP6*, *NMNAT1*, and *NADK* were correlated with the clinical stage (Fig. S5), suggesting their involvement in KIRC progression. Subsequently, a regulatory axis of ceRNA interactions was constructed to elucidate the potential molecular mechanisms of these five genes in KIRC. A total of 285 miRNAs predicted to interact with these genes were identified using mirTarBase and Starbase (Fig. 13A). Among these, hsa-miR-135a-5p, hsa-miR-4761-3p, hsa-miR-4735-5p, hsa-miR-4775, and hsa-miR-488-3p were designated as highly connected target mRNAs based on Cytohubba calculations (Fig. 13B). However, univariate Cox analysis indicated that only hsa-miR-135a-5p expression correlated with the survival probability of KIRC patients (Fig. 13C). Furthermore, KIRC patients exhibiting elevated hsa-miR-135a-5p levels demonstrated significantly poorer survival probabilities (*p* = 0.019). The lncRNA targets associated with hsa-miR-135a-5p were identified through StarBase to establish the miRNA-lncRNA axis (Fig. 13D). Kaplan–Meier analysis revealed that OIP5-AS1 expression significantly correlated with survival; patients with higher OIP5-AS1 levels had reduced survival probabilities (Fig. 13E). Consequently, the OIP5-AS1/hsa-miR-135a-5p/*ENPP3*/*NAMPT* regulatory axis and ceRNA network were preliminarily established, indicating their potential significance in KIRC progression.

## Discussion

NAM, a water-soluble amide form of vitamin B3 and a precursor to NAD^+^ (Jung et al., 2022), alongside its metabolic components (NAD^+^, NMN, and the key enzyme *NAMPT*), is essential for DNA repair, gene stability, and the regulation of the immune microenvironment (Audrito et al., 2011; Burgos, 2011; Garten et al., 2015; Travelli et al., 2019). NAD+ is an important coenzyme that functions as a universal electron acceptor in glycolysis and the tricarboxylic acid cycle. It is also a substrate or cofactor for many enzymes, such as PARP, sirtuins, and CD38, which are closely related to cellular metabolism (Verdin, 2015; Covarrubias et al., 2021). These three classes of NAD+-dependent enzymes, *i.e.,* NAD+-consuming enzymes, degrade NAD+ to produce the byproduct nicotinamide (NAM). Dysregulation of NAD+ levels is closely associated with metabolic disorders, neurodegenerative diseases, and the development of tumors (Verdin, 2015; Amjad et al., 2021). Preclinical studies have shown that decreased NAD+ levels are closely associated with kidney disease (Oh et al., 2014; Muraoka et al., 2019), and supplementation with NAD+ precursors is beneficial to the kidneys (Tran et al., 2016; Myakala et al., 2023; Muraoka et al., 2019; Yasuda et al., 2021; Guan et al., 2017), suggesting that NAD+ replacement therapy may be extended from rodent models to patients with kidney disease.

Recent research indicates that NAM supplementation effectively inhibits the progression of various malignancies, including breast cancer, chronic lymphocytic leukemia, and hepatocellular carcinoma (Audrito et al., 2011; Luo et al., 2001). In the context of renal tumorigenesis, NAM exhibits both tumor-suppressive and tumor-promoting properties. Rakieten et al. demonstrated that NAM inhibited renal tumor formation in streptozotocin-exposed animal models (Rakieten et al., 1976), whereas Rosenberg et al. found that it promoted renal tubular tumor development (Rosenberg et al., 1985). Despite these findings, the role of NAM metabolism in KIRC remains poorly understood. This study constructed NMRS for risk stratification, prognosis prediction, and transcriptional-based immunotherapy guidance for clinicians in managing KIRC patients.

With advancements in bioinformatics technology, many predictive gene signatures have emerged through various machine-learning algorithms. This study introduced a novel NAM-related gene signature developed *via* a combination of univariate and multivariate Cox models. This approach enables further dimensionality reduction of the variables, promoting the construction of a robust gene signature comprising five NAM-related genes.

This study identified five genes (*ENPP3*, *NADK*, *NAMPT*, *NMNAT1*, and *PARP6*) as potential prognostic features exhibiting strong predictive capability. In contrast to previous studies, our nomogram has a robust predictive accuracy (Dong et al., 2024; Zhang et al., 2022).

*ENPP3*, a member of the ENPP family, is vital in physiological processes, particularly in enhancing tumor cell motility and migration. Higher *ENPP3* has been reported in conditions such as clear cell renal cell carcinoma, cholangiocarcinoma, and colorectal cancer; thus, *ENPP3* may be a potential tumor marker (Qin et al., 2023; Deissler et al., 1999; Donate et al., 2016; Wu et al., 2019).

*NADK* is a critical enzyme that catalyzes the conversion of NAD^+^ to NADP^+^, serving as a significant source of NADPH. Elevated NADPH levels, mediated by *NADK*, play a vital role in the metastasis of breast cancer. Furthermore, pancreatic ductal adenocarcinoma (PDAC) also contributes to the regulation of increased *NADK* activity (Hoxhaj et al., 2019).

*NAMPT* is upregulated in various malignancies, including breast, colon, prostate, thyroid, and gastric cancers, as well as multiple hematopoietic malignancies. In specific tumors such as sarcomas, thyroid cancer, and prostate cancer, elevated *NAMPT* expression correlates with increased tumor invasion depth, metastatic potential, and chemoresistance (Sawicka-Gutaj et al., 2015; Vora et al., 2016; Shackelford et al., 2013). *NAMPT* exists in two forms: intracellular and extracellular, with the latter primarily produced by adipocytes and exhibiting higher enzymatic activity.

Shi et al. (2021) identified *NMNAT1* as a previously unrecognized genetically dependent gene in leukemia stem cells (LSCs), which prevents p53 activation by enhancing NAD+ biosynthesis.

*PARP6*, a recently identified member of the PARP family, is situated on chromosome 15q23 and comprises 630 amino acids. Despite its discovery, research on *PARP6* in malignant tumors remains limited. A prior study by Qi et al. (2016) in colorectal cancer revealed that *PARP6* regulated the survival of inhibitors of apoptosis protein family members, thereby influencing cancer cell progression (Tuncel et al., 2012).

In summary, the five selected NMRGs play significant roles in the regulation of metabolic processes in cancer, either directly or indirectly.

Clinical correlation analysis revealed a significant relationship between the risk score and key demographic and clinical parameters. Samples with higher tumor grade and TNM stage received higher risk scores. However, no significant age and gender-dependent risk stratification was observed.

This study investigated the biological mechanisms and immune infiltration characteristics associated with the identified gene signature. CIBERSORT and ssGSEA analyses revealed differences in immune cell expression between the two risk groups. Notably, the high-risk group exhibited a markedly low distribution of activated dendritic cells (DCs) and mast cells. Although DCs are infrequent in tumors and lymphoid organs, they play a critical role in initiating anti-tumor immunity. Consequently, the regulation of DCs is essential for inducing effective anti-tumor responses, suggesting potential new strategies for anti-tumor therapies.

Additionally, this study established a novel ceRNA network previously unreported in KIRC. High expression of lncRNA OIP5-AS1 was associated with poor prognosis. Consequently, the lncRNA OIP5-AS1/hsa-miR-135a-5p/ENNP3/*NAMPT* regulatory axis may play a role in inhibiting tumor progression in KIRC. We will further validate the accuracy of the ceRNA network using a luciferase reporter assay.

However, it must be recognized that this study has some limitations. As a retrospective study, some patients were lost to follow-up, resulting in the absence of some important clinical endpoints in certain cohorts. Even with rigorous criteria, selection bias was unavoidable. Before building the multivariate Cox model, we did not employ additional feature selection techniques such as Lasso regression, which might lead to a risk of overfitting. Furthermore, the mechanisms of action of the five candidate NMRGs and the ceRNA network we identified require further investigation *in vitro* and *in vivo*, and we are also conducting related basic experiments to explore their mechanisms and potential biological functions. *In vitro* investigations should utilize techniques including gene knockout, overexpression, and RNA interference to systematically pinpoint critical genes and clarify their oncogenic or tumor-suppressive roles. In parallel, well-designed *in vivo* studies—such as the establishment of mouse models like xenograft and spontaneous tumor models—are essential for evaluating the contributions of key genes to tumor initiation and progression, as well as their impact on survival and prognosis in mice.

### Conclusion

In summary, this study identified a novel NMRS through bioinformatics analysis, offering a predictive tool for KIRC prognosis. External datasets validated the model’s accuracy and compensated for the limitations of the study. Additionally, this gene signature demonstrates significant potential in predicting the immune microenvironment and immunotherapy responses, providing valuable insights for clinical management.

## Supplemental Information

10.7717/peerj.21300/supp-1 Supplemental Information 1 RT- PCR raw data

10.7717/peerj.21300/supp-2 Supplemental Information 2 Code

10.7717/peerj.21300/supp-3 Supplemental Information 3 Primer sequences employed by NAMPT, PARP6, and GAPDH

10.7717/peerj.21300/supp-4 Supplemental Information 4 NMRGs identified 42 NMRGs were identified using two NAM metabolism-related gene sets.

10.7717/peerj.21300/supp-5 Supplemental Information 5 ENPP3-Gefitinib data

10.7717/peerj.21300/supp-6 Supplemental Information 6 K-M curve of the relationship between OS in KIRC patients and expression levels of NMRGs (A–E)

10.7717/peerj.21300/supp-7 Supplemental Information 7 Relationship between the five NMRGs and clinicopathological factors (A–E)

10.7717/peerj.21300/supp-8 Supplemental Information 8 Development and assessment of prognostic signature based NMRGs in the ICGA date set (A–C) Risk score distribution (A), survival status of each patient (B), and Kaplan–Meier curves of low- and high-risk subgroups based on risk score (C). (D–F) Receiver operating characteristic analysis of KIRC risk scores and other prognostic clinical characteristics to predict the 1-, 3-, and 5-year survival rate of KIRC patients.

10.7717/peerj.21300/supp-9 Supplemental Information 9 Relationship between the Risk score and Drug sensitivity group among Sunitinib, Rapamycin, Pyrimethamine Paclitaxel, Lenalidomide, Imatinib, Gefitinib, Cytarabine, and Bosutinib (A)

10.7717/peerj.21300/supp-10 Supplemental Information 10 Relationship between ENPP3, NMNAT1, NAMPT,NADK, and PARP6 and clinical stages (A–E)

10.7717/peerj.21300/supp-11 Supplemental Information 11 MIQE checklist

## Additional Information and Declarations

### Data Availability

The following information was supplied regarding data availability: The raw data and code is available in the Supplemental Files. The raw data is available at Zenodo: - dong, . pei . (2025). Molecular dynamics [Data set]. Zenodo. https://doi.org/10.5281/zenodo.16730288
