# Supplementary material for: Nicotinamide metabolism-related signature and lncRNA regulatory network in kidney renal clear cell carcinoma
Source: PeerJ. 2026 Jun 9;14:e21300. doi: 10.7717/peerj.21300 (PMC13262547; doi:10.7717/peerj.21300)
Supplement: Supplemental Information 2 [file peerj-14-21300-s002.docx]

library(multiMiR)

setwd("D:\\美容基因\\肾透明细胞癌")

x = read.table("gene.txt",stringsAsFactors = F)

gene2mir <- get_multimir(org = 'hsa',

target = x,

table = "all",

summary = TRUE,

predicted.cutoff.type = 'n',

predicted.cutoff = 500000)

#table= "predicted"/'validated'

save(gene2mir, file = "gene2mir.Rdata")

ez = gene2mir@data[gene2mir@data$database=="mirtarbase",]

write.table(ez, file="mirtarbase.txt", sep="\t", quote=F, row.names=F)

library(estimate)

library(reshape2)

library(ggpubr)

library(Rmisc)

library(ggplot2)

library(ggunchained)

library(limma)

library(TCGAbiolinks)

library(e1071)

library(preprocessCore)

library(parallel)

library(RColorBrewer)

library(tidyr)

library(tidyverse)

install.packages('preprocessCore')

setwd("D:\\美容基因\\肾透明细胞癌")

rt=read.table("gene_all_fpkm.txt",header=T,sep="\t",check.names=F)

risk=read.table("risk.txt",header=T,sep="\t",check.names=F)

#读取输入文件

rt=as.matrix(rt)

rownames(rt)=rt[,1]

exp=rt[,2:ncol(rt)]

dimnames=list(rownames(exp),colnames(exp))

data=matrix(as.numeric(as.matrix(exp)),nrow=nrow(exp),dimnames=dimnames)

data=avereps(data)

data=data[rowMeans(data)>0,]

#正常和肿瘤数目

group=sapply(strsplit(colnames(data),"\\-"),"[",4)

group=sapply(strsplit(group,""),"[",1)

group=gsub("2", "1", group)

conNum=length(group[group==1]) #正常组样品数目

treatNum=length(group[group==0]) #肿瘤组样品数目

grade=c(rep(1,conNum), rep(2,treatNum))

conNum=72

treatNum=541

#重新排序data

##正常组织样本

SamN <- TCGAquery_SampleTypes(barcode = colnames(data),typesample = "NT")

##肿瘤组织样本

SamT <- setdiff(colnames(data),SamN)

data <- data[,c(SamN,SamT)]#重新排序一下列的顺序

write.table(data,"gene_all_fpkm_sort.txt",row.names=T,quote=F,sep="\t")

write.table(data[,73:ncol(data)],"gene_all_fpkm_tumor.txt",row.names=T,quote=F,sep="\t")

#数据转换

v <-voom(data, plot=F, save.plot=F)

out=v$E

out=rbind(ID=colnames(out), out)

write.table(out,file="uniq.symbol.txt",sep="\t",quote=F,col.names=F)

#运行CIBERSORT，得到免疫细胞浸润的结果

source("CIBERSORT.R")

results=CIBERSORT("lm22.txt", "uniq.symbol.txt", perm=1000, QN=TRUE)

#开始作图 合并risk

results=read.table("results2.txt",header=T,sep="\t",check.names=F)

risk=read.table("risk.txt",header=T,sep="\t",check.names=F)

risk1=risk[,1]

risk2=risk[,ncol(risk)]

risk3=cbind(risk1,risk2)

colnames(risk3)=c("NAME","risk")

samesample=intersect(as.vector(risk3[,1]),as.vector(results[,1]))

rownames(risk3)=risk3[,1]

rownames(results)=results[,1]

risk3=risk3[samesample,]

results=results[samesample,]

cibersort_risk=cbind(results,risk3[,2])

colnames(cibersort_risk)[ncol(cibersort_risk)]="risk"

write.table(cibersort_risk,row.names=F,"cibersort_risk.txt",quote=F,sep="\t")

#预处理

cibersort_risk$risk=factor(cibersort_risk$risk, levels=c("low","high"))

data=melt(cibersort_risk, id.vars=c("risk"))

data=data[519:nrow(data),]

colnames(data)=c("Risk", "scoreType", "Score")

data$Score=as.numeric(data$Score)

data=data[1:11396,]

#直方图

pdf(file="cibersortbox.pdf", width=22, height=10)

ggplot(dat,aes(Sample,Proportion,fill = Cell_type)) +

geom_bar(stat = "identity") +

labs(fill = "Cell Type",x = "",y = "Estiamted Proportion") +

theme_bw() + theme(text = element_text(size = 23))+

theme(axis.text.x = element_blank(),

axis.ticks.x = element_blank(),

legend.position = "bottom") +

scale_y_continuous(expand = c(0.01,0)) +

scale_fill_manual(values = mypalette(22))

dev.off()

library(tidyverse)

library(data.table)

library(GSVA)

library(limma)

library(ggsci)

library(tidyr)

library(ggpubr)

library(RColorBrewer)

library(corrplot)

setwd("C:\\r")

gsva_data =read.table("exp_T.txt",header=T,sep="\t",row.names=True,check.names=F)

expr=read.table("gene_all_fpkm_tumor.txt",header=T,sep="\t",check.names=F)

#相关性热图

resm <- gsva_data

for (i in colnames(gsva_data)) {

resm[,i] <- (gsva_data[,i] -min(gsva_data[,i]))/(max(gsva_data[,i] )-min(gsva_data[,i] ))

}

resmcor <- cor(resm)

resmorp <- cor.mtest(resmcor, conf.level = .95) #使用cor.mtest做显著性检验;

#提取p值矩阵；

p.mat <- resmorp$p

#go富集分析

kk <- enrichGO(gene = gene,

OrgDb = org.Hs.eg.db,

pvalueCutoff =0.05,

qvalueCutoff = 0.05,

ont="all",

readable =T)

write.table(kk,file="GO.txt",sep="\t",quote=F,row.names = F) #保存富集结果

ego=read.table("GO.txt", header = T,sep="\t",check.names=F) #读取kegg富集结果文件

go=data.frame(Category = "All",ID = ego$ID,Term = ego$Description, Genes = gsub("/", ", ", ego$geneID), adj_pval = ego$p.adjust)

#读取基因的logFC文件

#读取基因的logFC文件

id.fc <- read.table("id.txt", header = T,sep="\t",check.names=F)

id.fc=id.fc[is.na(id.fc[,"entrezID"])==F,] #去除基因id为NA的基因

genelist <- data.frame(ID = id.fc$gene, logFC = id.fc$logFC)

row.names(genelist)=genelist[,1]

circ <- circle_dat(go, genelist)

#GO圈图绘制

termNum = 20 #限定term数目

geneNum = nrow(genelist) #限定基因数目

chord <- chord_dat(circ, genelist[1:geneNum,], go$Term[1:termNum])

pdf(file="circ.pdf",width = 20,height = 15)

GOChord(chord,

space = 0.001, #基因之间的间距

gene.order = 'logFC', #按照logFC值对基因排序

gene.space = 0.25, #基因名跟圆圈的相对距离

gene.size = 4, #基因名字体大小

border.size = 0.1, #线条粗细

process.label = 7.5) #term字体大小

dev.off()

termCol <- c("#223D6C","#D20A13","#FFD121","#088247","#58CDD9","#7A142C","#5D90BA","#431A3D","#91612D","#6E568C","#E0367A","#D8D155","#64495D","#7CC767")

pdf(file="cluster.pdf",width = 20,height = 15)

GOCluster(circ.gsym,

go$Term[1:termNum],

lfc.space = 0.2, #倍数跟树间的空隙大小

lfc.width = 1, #变化倍数的圆圈宽度

term.col = termCol[1:termNum], #自定义term的颜色

term.space = 0.2, #倍数跟term间的空隙大小

term.width = 1) #富集term的圆圈宽度

dev.off()

#绘制GO气泡图

pdf(file="GOBubble2.pdf",width=25,height=35)

GOBubble(circ,labels=3,table.legend=F)

dev.off()

#绘制GO圈图

pdf(file="GOCircle.pdf",width=25,height=6)

GOCircle(circ,rad1=2.5,rad2=3.5,label.size=4,nsub=10)

#rad1外圈的注释；nsub=10中10代表显示GO的数据，可修改

dev.off()

#绘制GO热图

termNum = 20 #限定term数目

geneNum = nrow(genelist) #限定基因数目

chord <- chord_dat(circ, genelist[1:geneNum,], go$Term[1:termNum])

pdf(file="GOHeat.pdf",width = 30,height = 40)

GOHeat(chord, nlfc =1, fill.col = c('red', 'white', 'blue'))

dev.off()

setwd("D:\\美容基因\\肾透明细胞癌")

library("org.Hs.eg.db")

rt=read.table("diff.txt",sep="\t",check.names=F,header=T)

genes=as.vector(rt[,1])

entrezIDs <- mget(genes,org.Hs.egSYMBOL2EG, ifnotfound=NA)

#找出基因对应的id

entrezIDs <-as.character(entrezIDs)

out=cbind(rt,entrezID=entrezIDs)

write.table(out,file="id.txt",sep="\t",quote=F,row.names=F)

library("clusterProfiler")

library("org.Hs.eg.db")

library("enrichplot")

library(GOplot)

rt=read.table("id.txt",sep="\t",check.names=F,header=T)

rt=rt[is.na(rt[,"entrezID"])==F,] #去除基因id为NA的基因

gene=rt$entrezID

#kegg富集分析

R.utils::setOption("clusterProfiler.download.method",'auto')

kk <- enrichKEGG(gene = gene, organism = "hsa", pvalueCutoff =0.5, qvalueCutoff =0.5) #富集分析

write.table(kk,file="KEGGId.txt",sep="\t",quote=F,row.names = F) #保存富集结果

#文件

go=data.frame(Category = "All",ID = ego$ID,Term = ego$Description, Genes = gsub("/", ", ", ego$geneID), adj_pval = ego$p.adjust)

#读取基因的logFC文件

#读取基因的logFC文件

id.fc <- read.table("id.txt", header = T,sep="\t",check.names=F)

id.fc=id.fc[is.na(id.fc[,"entrezID"])==F,] #去除基因id为NA的基因

genelist <- data.frame(ID = id.fc$entrezID, logFC = id.fc$logFC)

row.names(genelist)=genelist[,1]

circ <- circle_dat(go, genelist)

#KEGG圈图绘制

termNum = 14 #限定term数目

geneNum = nrow(genelist) #限定基因数目

chord <- chord_dat(circ, genelist[1:geneNum,], go$Term[1:termNum])

pdf(file="circ.pdf",width = 20,height = 14)

GOChord(chord,

space = 0.001, #基因之间的间距

gene.order = 'logFC', #按照logFC值对基因排序

gene.space = 0.25, #基因名跟圆圈的相对距离

gene.size = 4, #基因名字体大小

border.size = 0.1, #线条粗细

process.label = 7.5) #term字体大小

dev.off()

termCol <- c("#223D6C","#D20A13","#FFD121","#088247","#58CDD9","#7A142C","#5D90BA","#431A3D","#91612D","#6E568C","#E0367A","#D8D155","#64495D","#7CC767")

pdf(file="cluster.pdf",width = 20,height = 9.6)

GOCluster(circ.gsym,

go$Term[1:termNum],

lfc.space = 0.2, #倍数跟树间的空隙大小

lfc.width = 1, #变化倍数的圆圈宽度

term.col = termCol[1:termNum], #自定义term的颜色

term.space = 0.2, #倍数跟term间的空隙大小

term.width = 1) #富集term的圆圈宽度

dev.off()

library(TCGAbiolinks)

query_miRNA = GDCquery(project = "TCGA-KIRC", experimental.strategy = "miRNA-Seq", data.category = "Transcriptome Profiling", data.type = "miRNA Expression Quantification", workflow.type = "BCGSC miRNA Profiling")

cellMarker =read.table("cellMarker.txt",header=T,sep="\t",check.names=F)

expr=read.table("gene_all_fpkm_tumor.txt",header=T,sep="\t",check.names=F)

#将cellMarker文件列名的第2个修改为celltype

colnames(cellMarker)[2] <- "celltype"

#将cellMarker文件以celltype为分组拆分成list数据格式

type <- split(cellMarker,cellMarker$celltype)

#将list中每个celltype中的基因进行合并

cellMarker <- lapply(type, function(x){

dd = x$Metagene

unique(dd)

})

save(cellMarker,file = "cellMarker_ssGSEA.Rdata")#保存中间文件

load("cellMarker_ssGSEA.Rdata")

rownames(expr) <- expr[,1] #将第一列作为行名

expr <- expr[,-1] #去除第一列

expr <- as.matrix(expr) #将expr转换为矩阵格式

dimnames=list(rownames(expr),colnames(expr))

expr=matrix(as.numeric(as.matrix(expr)),nrow=nrow(expr),dimnames=dimnames)

expr=avereps(expr)

expr=expr[rowMeans(expr)>0,]

#ssGSEA量化免疫浸润

gsva_data <- gsva(expr,cellMarker, method = "ssgsea")

a <- gsva_data %>% t() %>% as.data.frame()

write.table(a,"gsva_data.txt",quote=F,sep="\t")

a=read.table("gsva_data.txt",header=T,sep="\t",check.names=F)

#预处理

risk=read.table("risk.txt",header=T,sep="\t",check.names=F)

risk1=risk[,1]

risk2=risk[,ncol(risk)]

risk3=cbind(risk1,risk2)

colnames(risk3)=c("NAME","risk")

samesample=intersect(as.vector(risk3[,1]),as.vector(a[,1]))

rownames(risk3)=risk3[,1]

rownames(a)=a[,1]

risk3=risk3[samesample,]

a=a[samesample,]

ssgsea_risk=cbind(a,risk3[,2])

colnames(ssgsea_risk)[ncol(ssgsea_risk)]="group"

colnames(ssgsea_risk)[1]="sample"

write.table(ssgsea_risk,row.names=F,"ssgsea_risk.txt",quote=F,sep="\t")

ssgsea_risk <- as.matrix(ssgsea_risk)

ssgsea_risk[,2:29]=as.numeric(ssgsea_risk[,2:29])

ssgsea_risk=data.frame(ssgsea_risk)

ssgsea_risk$group=factor(ssgsea_risk$group, levels=c("low","high"))

axis.text.x = element_text(angle=45, hjust=1))

dev.off()

#相关性热图

resm <- gsva_data

for (i in colnames(gsva_data)) {

resm[,i] <- (gsva_data[,i] -min(gsva_data[,i]))/(max(gsva_data[,i] )-min(gsva_data[,i] ))

}

resmcor <- cor(t(resm))

resmorp <- cor.mtest(resmcor, conf.level = .95) #使用cor.mtest做显著性检验;

#提取p值矩阵；

p.mat <- resmorp$p

#相关性热图中展示显著性标记：

pdf(file="ssgsea_re.pdf", width=15, height=8)

corrplot(resmcor,

method = "color",

order = "hclust",

tl.cex = 1.0,

tl.col = "black",

p.mat = resmorp$p, sig.level = c(.001, .01, .05),outline="white",

insig = "label_sig",pch.cex = 1.0, pch.col = "white")

dev.off()

library(limma)

library(pheatmap)

library(TCGAbiolinks)

expFile="gene_all_fpkm_sort.txt" #表达输入文件

fdrFilter=0.05 #fdr临界值

logFCfilter=1 #logFC临界值

setwd("C:\\Users\\zlc\\Documents\\GDCdata\\TCGA-HNSC\\harmonized")

write.table(dat,"gene_all.txt",row.names=T,quote=F,sep="\t")

#写genemianyi_exp

rt=read.table("gene_all.txt",header=T,sep="\t",check.names=F,row.names=1)

rt=as.matrix(rt)

rt=rt[,-1]

rt=rt[,-2]

rownames(rt)=rt[,1]

gene=read.table("gene_meirong.txt",header=F,check.names=F,sep="\t")

sameGene=intersect(as.vector(gene[,1]),rownames(rt))

genemeirong_exp=exp[sameGene,]

write.table(genemeirong_exp,"gene_meirong.txt",row.names=T,quote=F,sep="\t")

#读取输入文件

exp=read.table(expFile, header=T, sep="\t",check.names=F)

exp=as.matrix(exp)

rownames(exp)=rt[,1]

rt=exp[,2:ncol(exp)]

dimnames=list(rownames(rt),colnames(rt))

data=matrix(as.numeric(as.matrix(rt)),nrow=nrow(rt),dimnames=dimnames)

data=avereps(data)

data=data[rowMeans(data)>0,]

#正常和肿瘤数目

group=sapply(strsplit(colnames(data),"\\-"),"[",4)

group=sapply(strsplit(group,""),"[",1)

group=gsub("2", "1", group)

conNum=length(group[group==1]) #正常组样品数目

treatNum=length(group[group==0]) #肿瘤组样品数目

grade=c(rep(1,conNum), rep(2,treatNum))

conNum=44

treatNum=522

#差异分析

outTab=data.frame()

for(i in row.names(data)){

geneName=unlist(strsplit(i,"\\|",))[1]

geneName=gsub("\\/", "_", geneName)

rt=rbind(expression=data[i,], grade=grade)

rt=as.matrix(t(rt))

wilcoxTest=wilcox.test(expression ~ grade, data=rt)

conGeneMeans=mean(data[i,1:conNum])

treatGeneMeans=mean(data[i,(conNum+1):ncol(data)])

logFC=log2(treatGeneMeans)-log2(conGeneMeans)

pvalue=wilcoxTest$p.value

conMed=median(data[i,1:conNum])

treatMed=median(data[i,(conNum+1):ncol(data)])

diffMed=treatMed-conMed

if( ((logFC>0) & (diffMed>0)) | ((logFC<0) & (diffMed<0)) ){

outTab=rbind(outTab,cbind(gene=i,conMean=conGeneMeans,treatMean=treatGeneMeans,logFC=logFC,pValue=pvalue))

}

}

pValue=outTab[,"pValue"]

fdr=p.adjust(as.numeric(as.vector(pValue)), method="fdr")

outTab=cbind(outTab, fdr=fdr)

#绘制差异基因热图1

geneNum=100

outDiff=outDiff[order(as.numeric(as.vector(outDiff$logFC))),]

diffGeneName=as.vector(outDiff[,1])

diffLength=length(diffGeneName)

hmGene=c()

if(diffLength>(2*geneNum)){

hmGene=diffGeneName[c(1:geneNum,(diffLength-geneNum+1):diffLength)]

}else{

hmGene=diffGeneName

}

hmExp=log2(data[hmGene,]+0.01)

Type=c(rep("Normal",conNum),rep("Tumor",treatNum))

names(Type)=colnames(data)

Type=as.data.frame(Type)

pdf(file="heatmap.pdf", height=7, width=10)

pheatmap(hmExp,

annotation=Type,

color = colorRampPalette(c(rep("blue",2), "white", rep("red",2)))(50),

cluster_cols =F,

show_colnames = F,

scale="row",

fontsize = 8,

fontsize_row=5,

fontsize_col=10)

dev.off()
