# Supplementary material for: Nicotinamide metabolism-related signature and lncRNA regulatory network in kidney renal clear cell carcinoma
Source: PeerJ. 2026 Jun 9;14:e21300. doi: 10.7717/peerj.21300 (PMC13262547; doi:10.7717/peerj.21300)
Supplement: Supplemental Information 3 [file peerj-14-21300-s003.docx]

NAMPT

Forward Sequence AATGTTCTCTTCACGGTGGAAAA

Reverse Sequence ACTGTGATTGGATACCAGGACT

PARP6

Forward Sequence GCCTATGGCAAAGGCATCTACC

Reverse Sequence TCTCTGGACCAGCTCATCCTTG

GAPDH：

 Forward Sequence CGCTGAGTACGTCGTGGAGTC

 Reverse Sequence GCTGATGATCTTGAGGCTGTTGTC
