# Supplementary figures and images for: Nicotinamide metabolism-related signature and lncRNA regulatory network in kidney renal clear cell carcinoma
Source: PeerJ. 2026 Jun 9;14:e21300. doi: 10.7717/peerj.21300 (PMC13262547; doi:10.7717/peerj.21300)

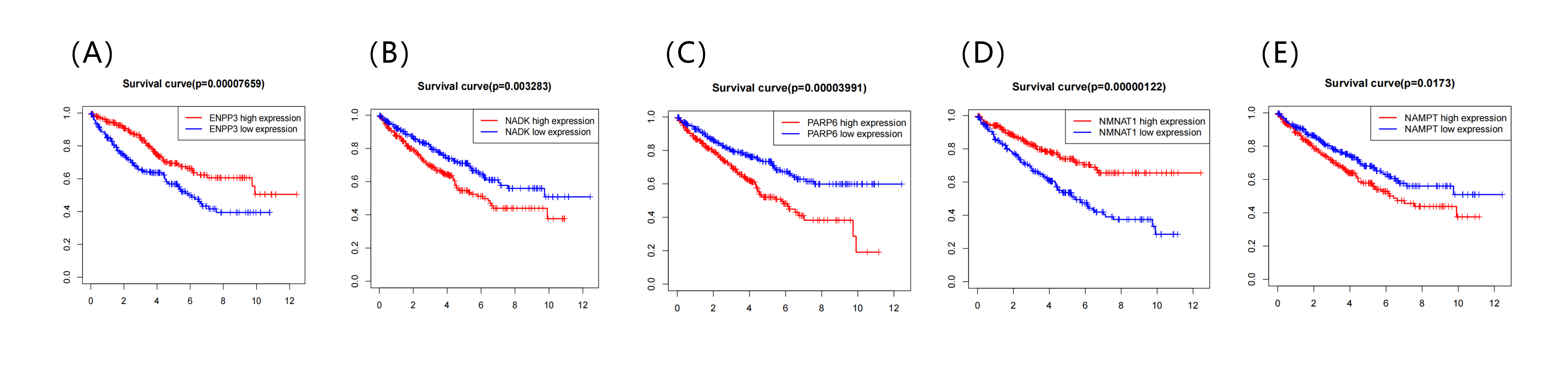

Supplement: Supplemental Information 6 [file peerj-14-21300-s006.png]

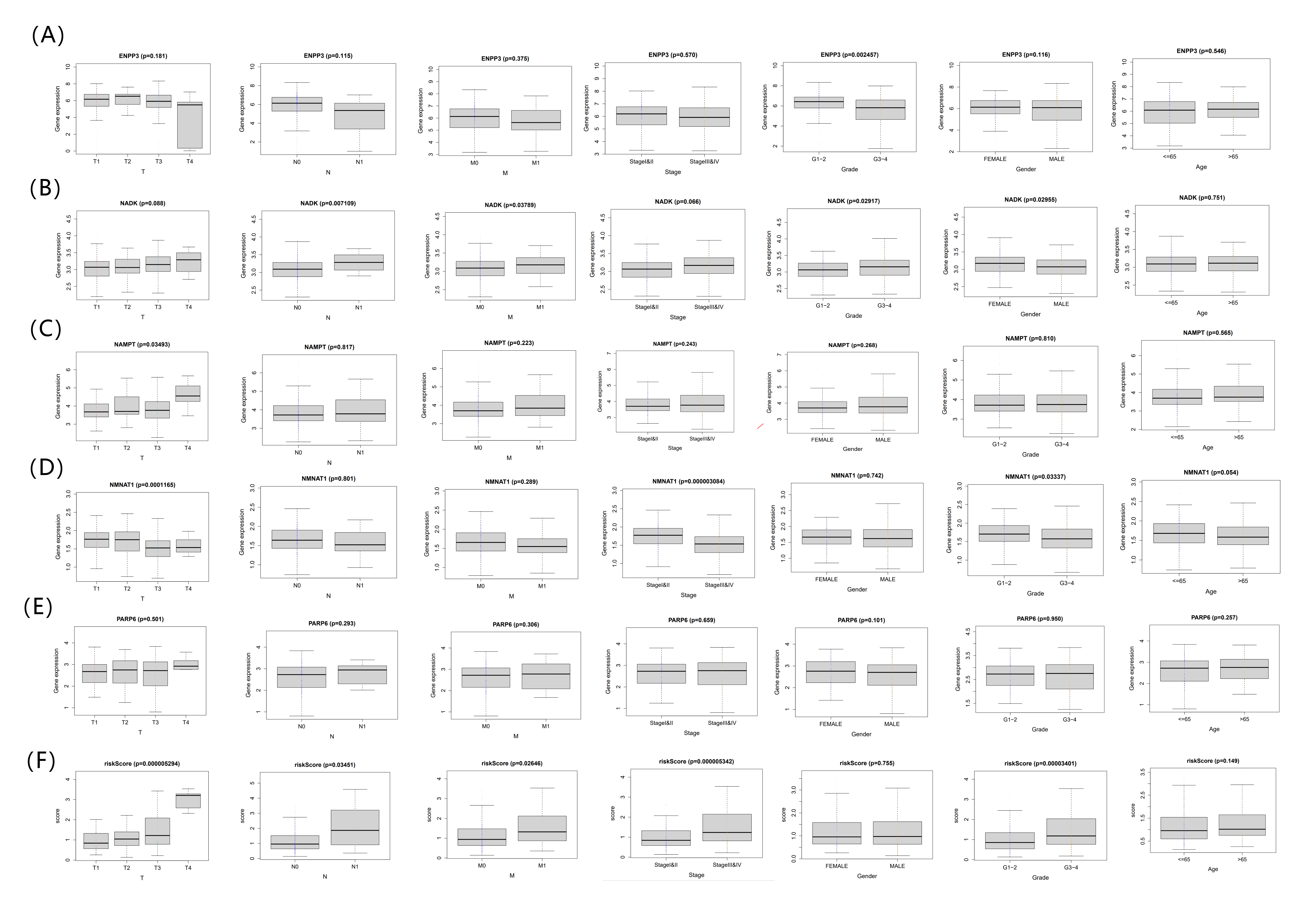

Supplement: Supplemental Information 7 [file peerj-14-21300-s007.png]

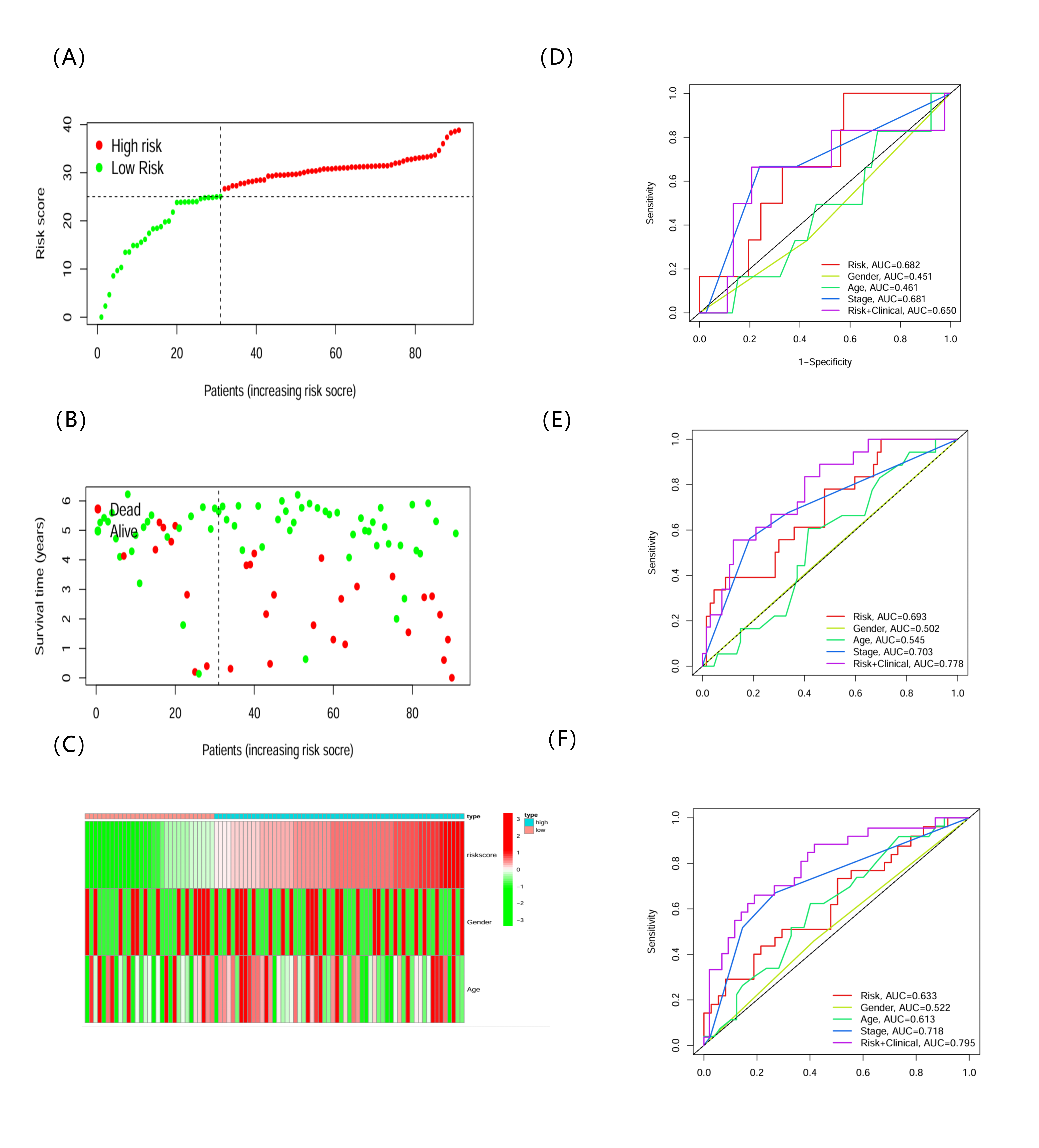

Supplement: Supplemental Information 8 — (A–C) Risk score distribution (A), survival status of each patient (B), and Kaplan–Meier curves of low- and high-risk subgroups based on risk score (C). (D–F) Receiver operating characteristic analysis of KIRC risk scores and other prognostic clinical characteristics to predict the 1-, 3-, and 5-year survival rate of KIRC patients. [file peerj-14-21300-s008.png]

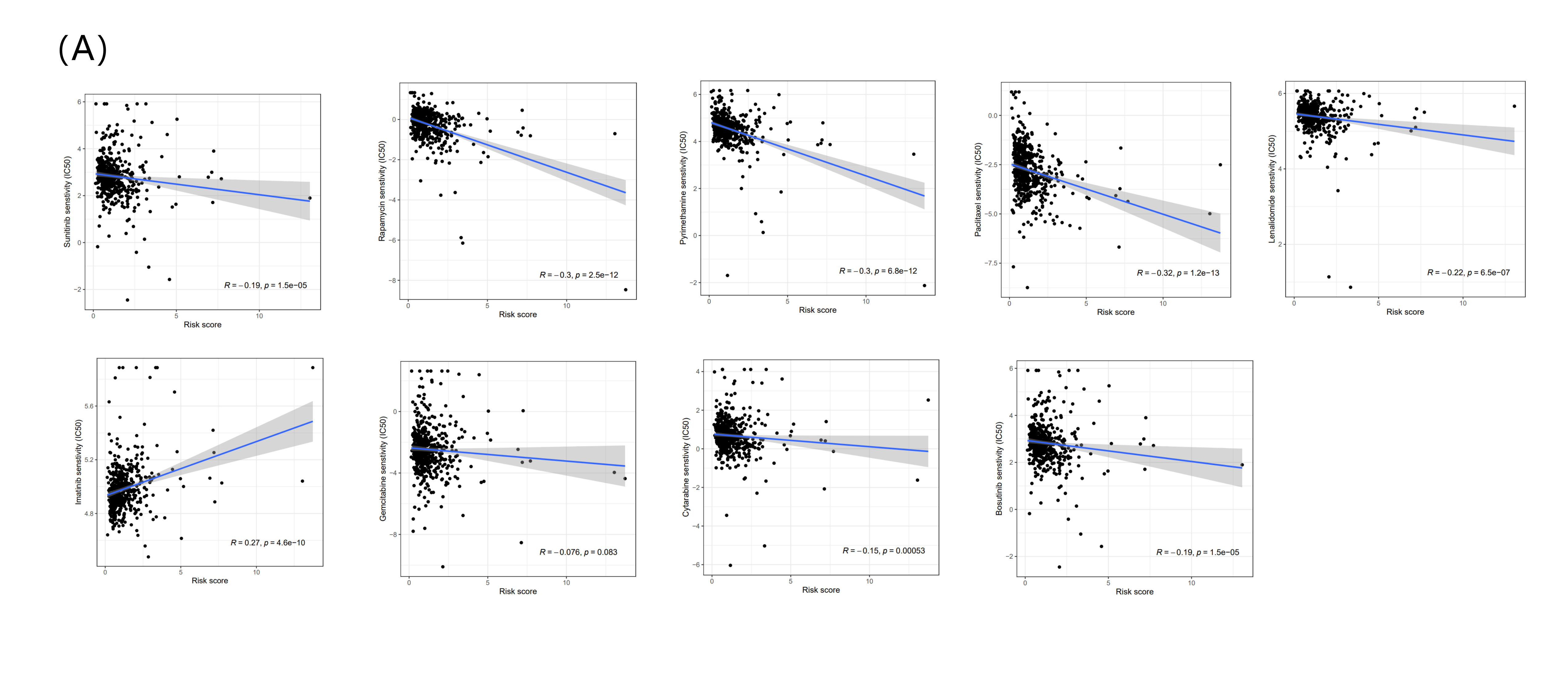

Supplement: Supplemental Information 9 [file peerj-14-21300-s009.png]

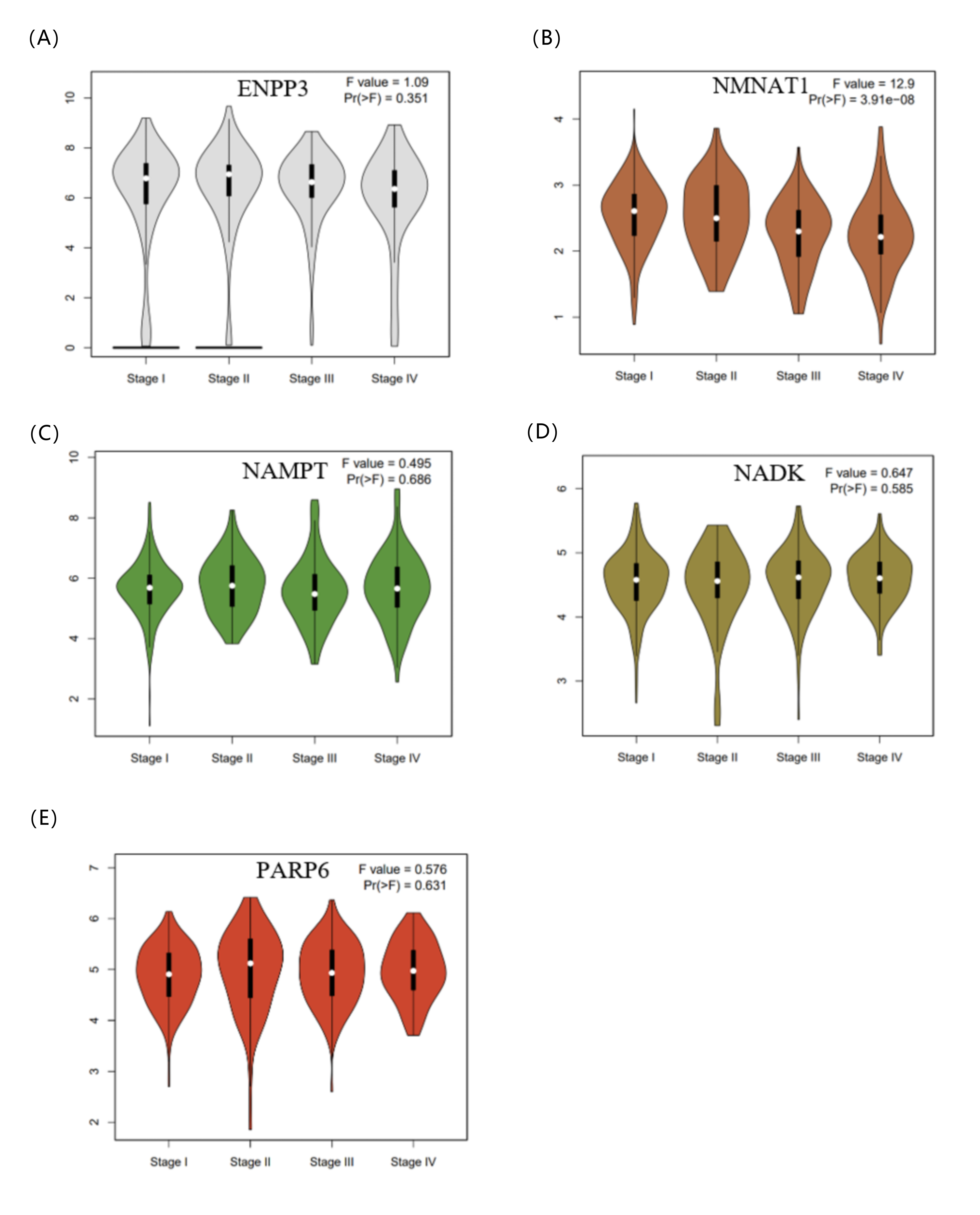

Supplement: Supplemental Information 10 [file peerj-14-21300-s010.png]
